# An organotypic atlas of human vascular cells

**DOI:** 10.1038/s41591-024-03376-x

**Published:** 2024-11-20

**Authors:** Sam N. Barnett, Ana-Maria Cujba, Lu Yang, Ana Raquel Maceiras, Shuang Li, Veronika R. Kedlian, J. Patrick Pett, Krzysztof Polanski, Antonio M. A. Miranda, Chuan Xu, James Cranley, Kazumasa Kanemaru, Michael Lee, Lukas Mach, Shani Perera, Catherine Tudor, Philomeena D. Joseph, Sophie Pritchard, Rebecca Toscano-Rivalta, Zewen K. Tuong, Liam Bolt, Robert Petryszak, Martin Prete, Batuhan Cakir, Alik Huseynov, Ioannis Sarropoulos, Rasheda A. Chowdhury, Rasa Elmentaite, Elo Madissoon, Amanda J. Oliver, Lia Campos, Agnieska Brazovskaja, Tomás Gomes, Barbara Treutlein, Chang N. Kim, Tomasz J. Nowakowski, Kerstin B. Meyer, Anna M. Randi, Michela Noseda, Sarah A. Teichmann

**Affiliations:** 1 National Heart and Lung Institute, Imperial College London, London, UK; 2 British Heart Foundation Centre of Research Excellence, Imperial College London, London, UK; 3 Wellcome Sanger Institute, Wellcome Genome Campus, Hinxton, UK; 4 Cambridge Stem Cell Institute and Department of Medicine, University of Cambridge, Cambridge, UK; 5 Ian Frazer Centre for Children’s Immunotherapy Research, Child Health Research Centre, University of Queensland, Brisbane, Queensland Australia; 6 Ensocell Therapeutics, BioData Innovation Centre, Wellcome Genome Campus, Cambridge, UK; 7 Max Planck Institute for Evolutionary Anthropology, Leipzig, Germany; 8 Department of Biosystems Science and Engineering, ETH Zürich, Basel, Switzerland; 9 Eli and Edythe Broad Center for Regeneration Medicine and Stem Cell Research, University of California, San Francisco, San Francisco, CA USA; 10 Weill Institute for Neurosciences, University of California, San Francisco, San Francisco, CA USA; 11 Department of Anatomy, University of California, San Francisco, San Francisco, CA USA; 12 Department of Psychiatry and Behavioral Sciences, University of California, San Francisco, San Francisco, CA USA; 13 Department of Neurological Surgery, University of California, San Francisco, San Francisco, CA USA; 14 Chan Zuckerberg Biohub, San Francisco, CA USA

**Keywords:** Computational biology and bioinformatics, Transcriptomics, Cell biology

## Abstract

The human vascular system, comprising endothelial cells (ECs) and mural cells, covers a vast surface area in the body, providing a critical interface between blood and tissue environments. Functional differences exist across specific vascular beds, but their molecular determinants across tissues remain largely unknown. In this study, we integrated single-cell transcriptomics data from 19 human organs and tissues and defined 42 vascular cell states from approximately 67,000 cells (62 donors), including angiotypic transitional signatures along the arterial endothelial axis from large to small caliber vessels. We also characterized organotypic populations, including splenic littoral and blood–brain barrier ECs, thus clarifying the molecular profiles of these important cell states. Interrogating endothelial–mural cell molecular crosstalk revealed angiotypic and organotypic communication pathways related to Notch, Wnt, retinoic acid, prostaglandin and cell adhesion signaling. Transcription factor network analysis revealed differential regulation of downstream target genes in tissue-specific modules, such as those of FOXF1 across multiple lung vascular subpopulations. Additionally, we make mechanistic inferences of vascular drug targets within different vascular beds. This open-access resource enhances our understanding of angiodiversity and organotypic molecular signatures in human vascular cells, and has therapeutic implications for vascular diseases across tissues.

## Abstract (web summary)

A vascular cell atlas integrating single-cell data of 19 organs and tissues from 62 donors identifies angiotypic and organotypic characteristics of endothelial and mural cells.

## Main

Blood vessel networks form the circulatory infrastructure of the body, comprising a hierarchical system of arteries, capillaries and veins that permeate tissues. This complex network regulates tissue homeostasis and exhibits functional heterogeneity beyond their vital role in delivery of blood flow for gas and nutrient exchange^1^. In parallel, the lymphatic vasculature removes interstitial fluid from tissue, which is transported and filtered through lymph nodes and is returned to the blood circulation. Crucially, vessels are involved in the pathogenesis of the vast majority of diseases, including hypertension, cancer, inflammatory disorders and diabetes.

Endothelial cells (ECs) are specialized cells that line the lumen of blood and lymphatic vessels, 95% of which are derived from capillaries reaching nearly every cell of organs and tissues within the human body^2^. ECs contribute to vital functions, including coagulation, immune regulation, angiogenesis and tissue repair^1^. Furthermore, beyond morphological distinctions in continuous, fenestrated and sinusoidal endothelium, ECs show organotypic functions, such as the blood–brain barrier, blood waste filtration (kidney glomeruli) and erythrocyte filtration (splenic littoral cells)^3^. These diverse roles represent inter-organ and intra-organ heterogeneities that are yet to be fully characterized at the molecular level.

Mural cells, including vascular smooth muscle cells (VSMCs) and pericytes, reside on the outer layer of vessels and have different morphologies and functions along the arteriovenous axis, with multi-layered VSMC coats around arteries and veins, and single-layer longitudinal or stellate pericytes around microvessels^4^. VSMCs regulate blood pressure and flow via their contractile function, and pericytes control microvascular homeostasis and angiogenesis. Although tissue-specific functions are well recognized for pericytes, recent single-cell studies have also demonstrated organotypic transcriptional diversity in mouse VSMCs^4,5^.

The molecular underpinnings of angiotypic differences and organotypic specializations of human endothelial and mural cells, however, remain incompletely characterized. Here we describe vascular cell diversity across 19 human organs and tissues. By defining 42 vascular cell populations, we highlight tissue-specific and shared gene signatures, regulatory networks, predicted druggable vascular cell targets, and intercellular signaling governing angiotypic and organotypic endothelial–mural cell interplay, creating a framework to advance our knowledge of physiology, pathology and potential therapeutic interventions.

## Results

### An integrated multi-organ map of the human vascular system

**Fig. 1:**
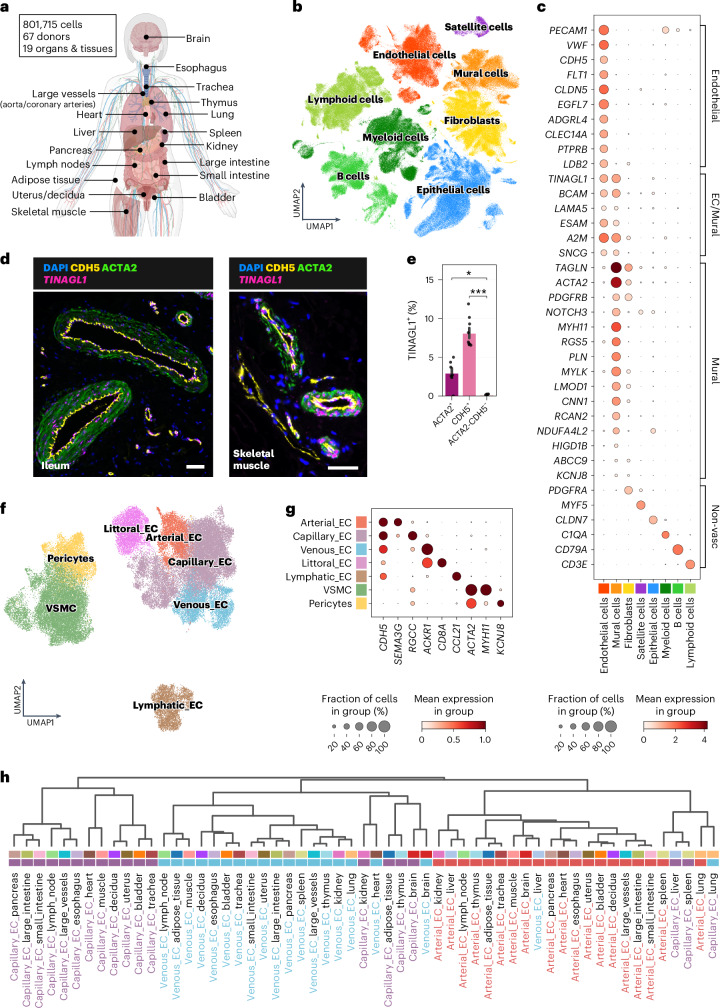
Overview of multi-organ vascular cell atlas. **a**, Organ and tissue single-cell RNA-seq datasets used for analysis. **b**, UMAP representation of all cell types from the global integrated object, including vascular and non-vascular types. **c**, Dot plot representation of selected EC and mural cell marker genes and markers of other major cell types identified in the global cell type object. **d**,**e**, smFISH for *TINAGL1* (scale bar, 50 μm) in ileum and skeletal muscle with nuclear counterstain (DAPI) (**d**) and quantification of *TINAGL1* co-expression with ACTA2 (VSMC) and CDH5 (EC) (*n* = 3 regions of interest (ROIs) × 3 donors) (**e**). Statistical analysis was performed using the Wilcoxon rank-sum test with Benjamini–Hochberg adjustment. * adjusted *P* < 0.05; *** adjusted *P* < 0.001. Error bars indicate standard error. **f**, UMAP representation of broad vascular cell types subset from the global integrated object. **g**, Dot plot representation of selected marker genes for major cell states within the vascular compartment. **h**, Dendrogram of hierarchical clustering of EC populations subset per organ. Top color bar: organ. Bottom color bar: EC subtype. The illustration in **a** was created with BioRender.

To characterize vascular cell heterogeneity in healthy human adults, we integrated single-cell datasets from 19 organs and tissues (Fig. 1a and Supplementary Tables 1 and 2). After quality control (QC), the global object comprised approximately 800,000 (67 donors, 166 samples) (Fig. 1b, Supplementary Data Figs. 1 and 2 and Supplementary Table 3). Data integration was performed using single-cell variational inference (scVI), without correcting for organ variation (Supplementary Data Fig. 2 and Methods). We identified major cell types, including endothelial, mural, epithelial, fibroblast, immune and muscle satellite cells^6^ (Fig. 1b,c, Supplementary Data Fig. 3 and Supplementary Table 4). ECs were defined by expression of *CDH5*, *VWF* and *PECAM1*, the latter also being detected in selected immune populations. Other broad EC markers included the angiogenesis-related *EGFL7* (ref. ^7^); the Wnt inhibitor *TMEM88*, which regulates angioblast differentiation during development^8^; and c-type lectin *CLEC14A*, considered a potential target for tumour angiogenesis^9^ (Fig. 1c and Supplementary Data Fig. 4). Mural cells expressed VSMC and pericyte markers *PDGFRB*, *NOTCH3*, *ACTA2*, *MYH11* and *RGS5*, in addition to contraction regulatory markers myosin light chain kinase (*MYLK*) and leiomodin-1 (*LMOD1*)^10^.

**Extended Data Fig. 1:**
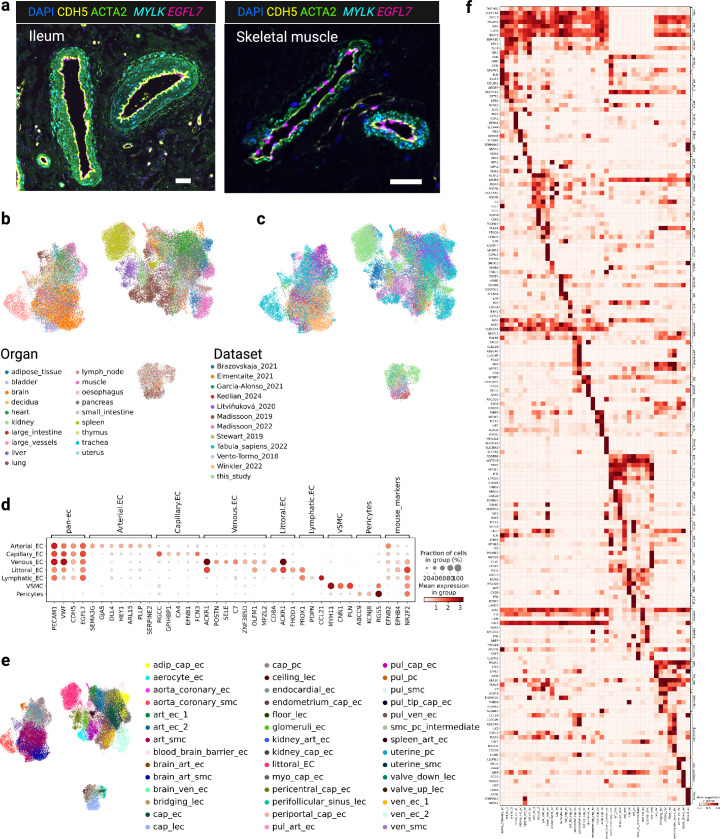
Integration of scRNA- seq data and vascular cell type markers. **a**, smFISH staining for EC markers CDH5 and *EGFL7*, as well as mural cell markers ACTA2 and *MYLK* in ileum and skeletal muscle tissue. Nuclei were counterstained with DAPI. Scale bars = 50 μm. **b**, UMAP representation of integrated organ datasets. **c**, UMAP representation of integrated individual datasets. **d**, Dotplot representation of vascular cell type gene markers. Fraction of expressing cells and average expression within each cell type is indicated by dot size and colour, respectively. **e**, UMAP representation of all endothelial and mural cell states. **f**, Heatmap representation of marker genes of all 42 vascular cell states identified in this study, with colour indicating mean gene expression per cell type.

Shared signatures between ECs and mural cells were also identified, including the integrin and EGFR binding molecule encoded by *TINAGL1* (Fig. 1c), which contributes to vessel formation in vitro^11^, as well as *ESAM* (endothelial cell-specific adhesion molecule). Furthermore, the immunoglobulin receptor, basal cell adhesion molecule (encoded by *BCAM*), and its binding partner laminin-α5 (encoded by *LAMA5*)^12^ were also shared (Fig. 1c), suggestive of common adhesion signatures across vascular cells. Validation using single-molecule fluorescence in situ hybridization (smFISH) confirmed *EGFL7* localization to ECs, *MYLK* in VSMCs and *TINAGL1* expression in both (Fig. 1d,e and Extended Data Fig. 1a).

To explore the heterogeneity of vascular cells, we subset and integrated the endothelial and mural compartments, obtaining approximately 67,000 cells from 62 donors, and identifying arterial, capillary, venous and lymphatic ECs, pericytes and VSMCs^13^ (Fig. 1f,g, Extended Data Fig. 1b–d and Supplementary Table 5). In addition to classic transcriptional signatures to differentiate arterial, venous and capillary ECs, we also highlighted alternative gene markers. Notably, the transcriptional signature of littoral ECs from splenic venous sinusoids were profiled at single-cell resolution (Fig. 1g and Extended Data Fig. 1d).

Consistent with previous findings, typical markers differentiating mouse arterial (*Efnb2)* and venous (*Ephb4*, *Vwf* and *Nr2f2)* ECs show little angiotypic specificity in human^14–16^ (Extended Data Fig. 1c), supporting the importance of human cell references. Other genes show specificity across both species, including *Sema3g*/*SEMA3G* in arterial ECs^14^.

To determine the similarity of ECs from different vascular beds, we performed hierarchical clustering on arterial, capillary and venous ECs by organ. This showed that phenotypes largely correlated with angiotypic identity (artery versus capillary versus vein) (Fig. 1h), contradicting previous reports for mouse ECs, where organotypic vascular cells (from the same organ/tissue) largely clustered together irrespective of their angiotypic identity^17^.

### Arterial ECs exhibit transitional signatures

We next explored EC populations at fine resolution, characterizing 42 angiotypic and organotypic vascular cell states (Extended Data Fig. 1e,f, Supplementary Data Fig. 5 and Supplementary Table 6).

**Fig. 2:**
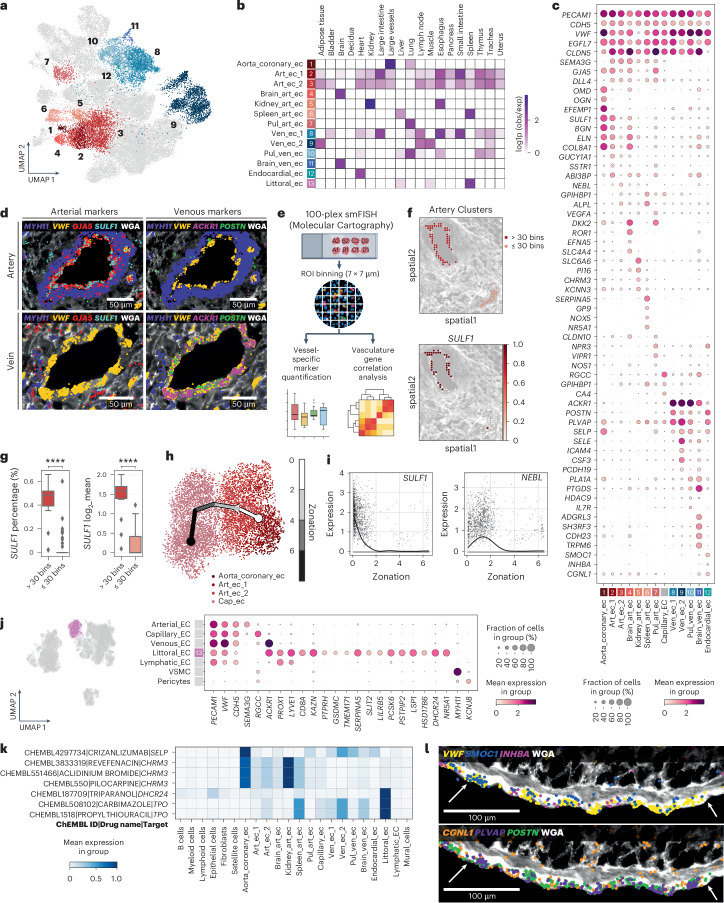
Endothelial heterogeneity in arteries and veins across tissues. **a**, UMAP representation of arterial, venous and endocardial cell states identified across blood ECs from all tissues. **b**, Heatmap representation of cell state enrichment per tissue. **c**, Dot plot representation of transcriptional signatures of arterial, venous and endocardial EC states. **d**, Multiplexed smFISH data visualization of arterial EC (*GJA5*, *SULF1*) and venous EC (*ACKR1*, *POSTN*) markers in an artery and vein. *MYH11* was used as a VSMC marker, and *VWF* as an EC marker. Tissues were counterstained with wheat germ agglutinin (WGA) to delineate cell membranes. **e**, Schematic for binning individual ROIs and downstream analysis. **f**, Expression of *SULF1* in arteries of different caliber using multiplexed smFISH. Top, spatial plot showing two arteries. Bottom, *SULF1* expression within different arterial vessels. Color bar represents gene counts present (1) or absent (0) in individual bins. **g**, Box plot representation of *SULF1* expression within larger (>30 bins) and smaller (≤30 bins) caliber arteries across all ROIs captured using multiplexed smFISH. Left, percentage of bins expressing *SULF1*. Right, mean expression of *SULF1* (expressing bins only). *n* = 90 vessels, 8 sections, 1 donor. Statistical analysis was performed using the Wilcoxon rank-sum test with Benjamini–Hochberg adjustment. **** adjusted *P* < 0.0001. For box plots in **g**, the center line shows the median; the box limits represent the 25th and 75th percentiles; the whiskers show the minimum and maximum values; and the dots represent potential outliers. **h**, Trajectory inference showing zonation patterning of arterial and capillary populations. **i**, Expression of *SULF1* and *NEBL* along the inferred zonation axis. **j**, UMAP (left) highlights the littoral EC cluster, and dot plot (right) representations selected marker genes of littoral ECs. **k**, Matrix plot visualization of cell state targets of drugs identified using drug2cell. **l**, Multiplexed smFISH data visualization of EC marker *VWF*, and *SMOC1*, *INHBA*, *CGNL1*, *PLVAP* and *POSTN* expression in endocardial ECs. White arrows indicate the endocardial layer. Scale bars, 100 µm. The illustration in **e** was created with BioRender.

Within arterial ECs, we identified three angiotypic cell states, including a cluster derived from aorta and coronary arteries (aorta_coronary_ec) and two populations shared across tissues (art_ec_1 and art_ec_2) (Fig. 2a,b). Aorta_coronary_ec and art_ec_1 expressed *BGN*, *ELN* and *SULF1*, encoding extracellular matrix (ECM) components and regulators contributing to vessel wall structure and stability in arteries exposed to high pressure (Fig. 2c). Furthermore, art_ec_1 and art_ec_2 showed expression of vascular homeostasis regulators from the Notch pathway, *HEY1* and *DLL4* (ref. ^18^), and *SEMA3G*. Conversely, aorta_coronary_ec enriched for other ECM protein-encoding genes, such as small leucine-rich proteoglycans (SLRPs) *OGN* and *OMD*, and the glycoprotein *EFEMP1*. SLRPs and glycoproteins are important in the basal lamina of blood vessels to facilitate cell–matrix interactions and provide structural support to withstand transmural pressures^19,20^. Furthermore, art_ec_2 expressed *NEBL* and the capillary marker *GPIHBP1*, suggesting an intermediate arteriolar phenotype (Fig. 2c).

**Extended Data Fig. 2:**
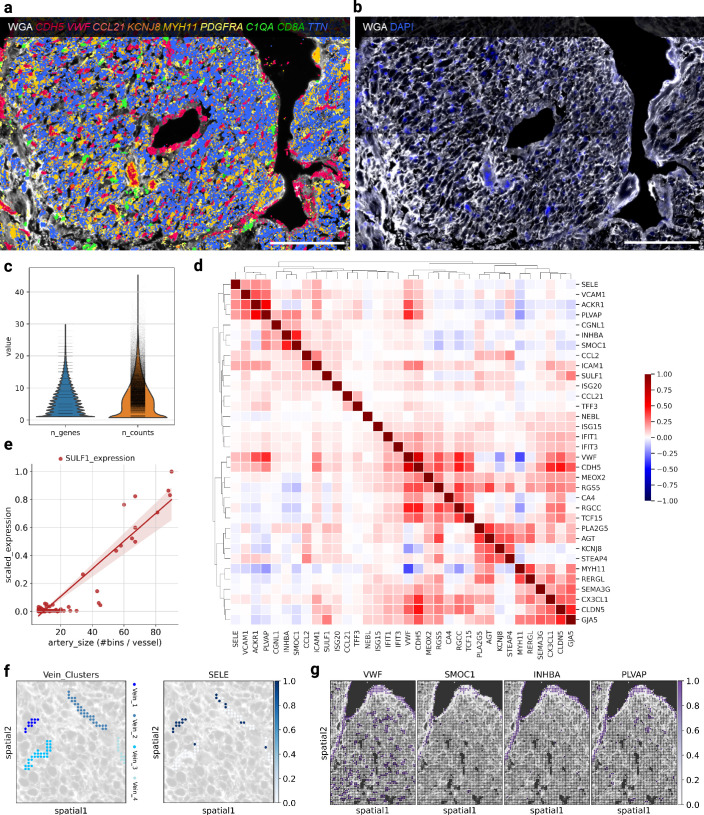
Characterisation of vascular EC using multiplexed-smFISH (Molecular Cartography^TM^). **a**, Multiplexed-smFISH visualisation of major cell type gene markers, including ECs (*CDH5*, *VWF*, *CCL21*), VSMC and pericytes (*MYH11* and *KCNJ8*, respectively), cardiomyocytes (*TTN*), fibroblasts (*PDGFRA*), myeloid cells (*C1QA*) and lymphoid cells (*CD8A*). **b**, WGA / DAPI staining for cell membranes and nuclei, respectively, on sections used for multiplexed-smFISH. Scale bars in (**a**) and (**b**) represent 200μm. **c**, Violin plot representation of n_genes/bin and n_counts/bin. **d**, Correlation heatmap with dendrogram of gene markers of vascular cells used for multiplexed- smFISH. Scale bar indicates correlation value calculated using Pearson correlation. Red = positive correlation. Blue = negative correlation. White = no correlation. **e**, Correlation of *SULF1* scaled expression by arterial vessel size (#bins / vessel). **f**, Subsetting of individual venous clusters and spatially resolved expression of venous marker *SELE*. Colour bar represents scaled gene counts per bin. **g**, Spatially resolved expression of endocardial markers *VWF*, *SMOC1*, *INHBA* and *PLVAP*. Colour bar represents scaled gene counts per bin.

**Extended Data Fig. 3:**
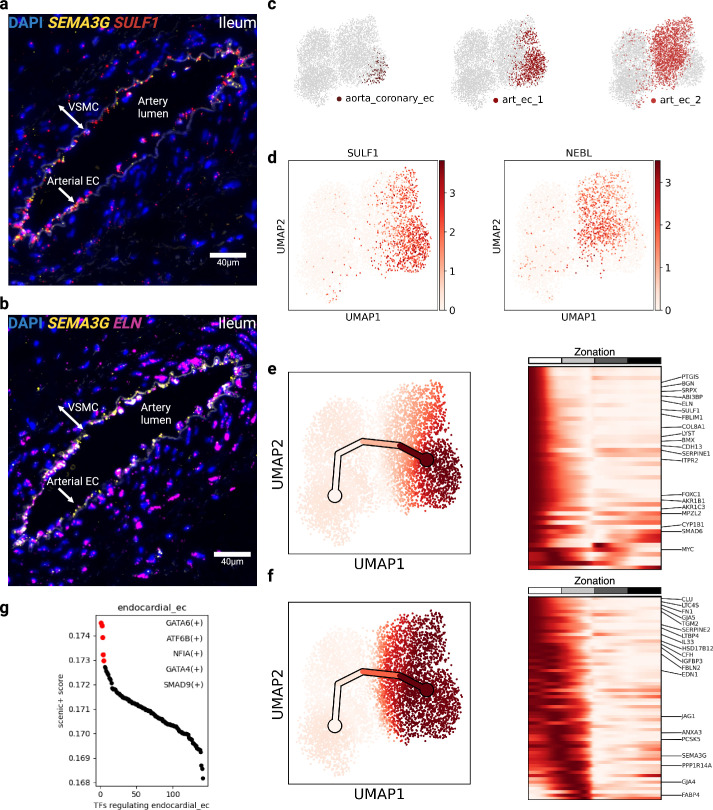
Characterisation of arterial, endocardial and capillary EC subpopulations. **a**, smFISH staining for *SULF1* (red - top panel) and **b**, *ELN* (magenta - bottom panel) with *SEMA3G* (yellow) in arterial EC. Nuclei were counterstained with DAPI. Scale bars = 40 μm **c**, UMAP representation of individual arterial EC populations after integration for trajectory inference (as shown in Fig. 2h). **d**, UMAP representation of *SULF1* (left) and *NEBL* (right) gene expression on integrated object used for trajectory inference. Scale bar represents log1p transformed gene expression values. **e** & **f**, UMAPs (left) show specific zonation signatures, and heatmaps (right) represent differentially expressed genes determined within specific zonation segments, including aorta_coronary_ec to art_ec_1 (**e**) and art_ec_1 to art_ec_2 (**f**). **g**, Regulon plots showing the SCENIC ranking of transcription factors for endocardial ECs with the top 5 transcription factors highlighted.

To understand if these three arterial EC states correlate to arterial size, we used high-resolution, highly multiplexed smFISH (Extended Data Fig. 2a,b and Supplementary Table 7). Qualitative exploration and gene correlation at subcellular level (7 × 7-µm bins) confirmed specific expression of *SULF1* in arterial ECs (*GJA5* and *SEMA3G*) and its absence in venous ECs (*ACKR1*, *SELE* and *PLVAP*^13^) (Fig. 2d–f and Extended Data Fig. 2c,d). Furthermore, clustering of bins to identify and classify individual arteries (*n* = 90) to larger and smaller vessels (>30 and ≤30 bins, respectively) revealed an increased percentage and mean expression of *SULF1* in larger arteries (Fig. 2f,g and Extended Data Fig. 2e). We also used low-plex smFISH to confirm co-expression of *ELN* and *SULF1* in arterial ECs (Extended Data Fig. 3a,b). To demonstrate further that the three arterial EC states exist along a zonation axis, trajectory inference was performed, which demonstrated a zonation from aorta_coronary_ec to art_ec_1, art_ec_2 and capillary_ec, associated with modules of differentially expressed genes (Fig. 2h,i and Extended Data Fig. 3c–f). This included decreased *SULF1* expression along the zonation axis, whereas *NEBL* specifically peaked in art_ec_2. These data suggest that arterial ECs have shared signatures across organs and distinct markers along the arterial axis.

### Organotypic specificity of arterial ECs

Four organotypic arterial EC states were enriched in the lung (pul_art_ec), brain (brain_art_ec), kidney (kidney_art_ec) and spleen (spleen_art_ec) (Fig. 2a,b).

Pul_art_ec expressed genes encoding vasodilation mediators, including *NPR3* and *VIPR1*, and *NOS1*, encoding a synthase enzyme for the vasodilator nitric oxide^21^ (Fig. 2c). These data align with the low pressure and resistance of the pulmonary circulation^22^. *DKK2*, a Wnt inhibitor, was expressed by pul_art_ec and in the brain counterpart (brain_art_ec)^23,24^. This latter population also expressed *ROR1*, encoding a non-canonical Wnt receptor^25^, consistent with the crucial role of Wnt signaling in development and homeostasis of the brain vasculature. The bicarbonate transporter encoded by *SLC4A4*, previously reported in astrocytes, also mapped to brain_art_ec^26^.

Kidney_art_ec expressed efferent arteriole marker *SLC6A6* (ref. ^27^), shear stress and vascular permeability regulator *PI16* (refs. ^28,29^) and vasodilation regulators *CHRM3* and *KCNN3* (refs. ^30,31^) (Fig. 2a–c). This signature is suggestive of fine-tuned vascular tone regulation required for blood filtration in the glomeruli.

The main feature of spleen_art_ec included enrichment of coagulation factor genes, including *SERPINA5* (protein C inhibitor) and *GP9* (glycoprotein IX)^32–34^, and *NOX5*, refining previous reports of NOX5 expression in human splenic ECs (Fig. 2b,c)^35^.

### Venous ECs display shared and organotypic signatures

We characterized four venous EC populations, one of which (ven_ec_1) was present across most organs and expressed *ACKR1*, *POSTN* and *PLVAP*^13,16^ (Fig. 2a–c). A second venous EC subtype (ven_ec_2) was enriched in skeletal muscle, adipose tissue and lymph node, and expressed *ICAM4* and *SELE*, encoding leukocyte adhesion molecules and the pro-inflammatory *IL6* (Fig. 2a–c). Thus, this endothelium is likely primed for immune activation and/or trafficking, similar to immune-recruiting venular ECs^36^. Two organotypic venous ECs were also identified (Fig. 2c). A lung-enriched population (pul_ven_ec) expressed *HDAC9* and *IL7R* and was negative for *COL15A1*, as previously described^23^. These shared expression of prostaglandin-related *PTGDS* and *PLA1A* with brain-enriched venous ECs (brain_ven_ec), which were also characterized by the G-protein-coupled receptor *ADGRL3*, and *SH3RF3*, associated with late-onset Alzheimer’s disease in genome-wide association studies (GWASs)^37^.

### Lymphatic ECs display organotypic features in lymph nodes

**Extended Data Fig. 4:**
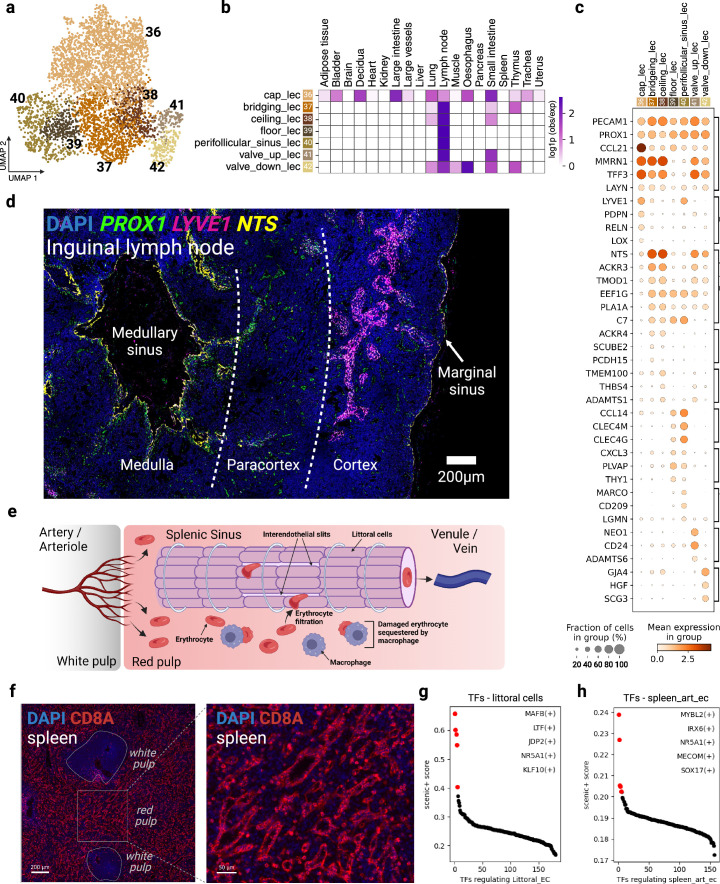
EC heterogeneity of lymphatics and lymphoid organs. **a**, UMAP representation of lymphatic EC states. **b**, Heatmap representation of cell state enrichment per tissue. **c**, Dotplot representation of lymphatic EC state gene markers. Fraction of expressing cells and average expression within each cell type is indicated by dot size and colour, respectively. **d**, smFISH representation of expression of *PROX1*, *LYVE1* and *NTS* in inguinal lymph node tissue. Nuclei were counterstained with DAPI. Scale bar = 200 μm. **e**, Schematic representation of splenic circulation. **f**, Immunostaining (Rarecyte) for CD8A in spleen tissue. DAPI was used for nuclei counterstaining. Scale bar = 200 um (left) and 50 um (right). **g** & **h**, Regulon plots showing the SCENIC ranking of transcription factors for littoral cells (**g**) and splenic arterial EC (**h**), with the top 5 transcription factors highlighted. The illustration in (**e**) was created with BioRender.com.

Lymphatic ECs (LECs), differing from their blood endothelial counterparts by carrying lymph fluid, displayed a unique signature including *PROX1*, *LYVE1* and *PDPN*. LECs comprised seven subpopulations, one of which, lymphatic capillary ECs (cap_lec), was enriched across organs (Extended Data Fig. 4a–c). *TFF3*, encoding the secretory protein trefoil factor 3 and implicated in lymphovascular invasion in breast cancer^38^, was expressed in cap_lec across all organs. Thus, increased levels of *TFF3* should be explored as a potential predictor for metastasis. The other LEC clusters predominantly corresponded to subtypes of lymph node infrastructure (Extended Data Fig. 4a–d), as described previously^39^.

### Littoral ECs share venous and lymphatic EC signatures

The spleen vasculature comprises an open circulation, where open-ended arterioles feed into the red pulp and erythrocytes are filtered through splenic venous sinuses^3,40^. These comprise morphologically elongated littoral ECs with phagocytic properties, characterized by expression of pan-EC markers *PECAM1* and *VWF*, as well as previously described T cell receptor *CD8A* and *FHOD1*, consistent with previous immunostaining and qRT–PCR data (Fig. 2j and Extended Data Fig. 4e,f)^41,42^. Co-expression of venous EC marker *ACKR1*, as well as lymphatic EC markers *PROX1* and *LYVE1*, suggests a hybrid venous-lymphatic phenotype.

SCENIC^43^ was used to predict cell-state-specific transcription factors and downstream target genes in splenic ECs. *NR5A1* (steroidogenic factor 1) emerged as a top transcription factor in littoral_ec and spleen_art_ec (Fig. 2c,j and Extended Data Fig. 4g,h). Notably, *NR5A1* is essential for splenic vasculature development and erythrocyte filtration in mice^44^, and mutations cause asplenia in humans^45^, implying a key role in splenic vasculature homeostasis. Littoral cell transcription factors also included *JDP2*, encoding an NR5A1 binding protein^46^, and *MAFB* (Extended Data Fig. 4g), which is typically expressed in tissue-resident macrophages^47^, consistent with the phagocytic-like phenotype of littoral cells. These results highlight organotypic signatures among vascular cells in the spleen.

### Endocardial ECs are an organotypic population of the heart

Lining the cardiac chambers, the endocardium (endocardial_ec) is crucial for valve and trabeculae formation^48^. Here we confirm expression of markers *SMOC1* and *INHBA*^49^, as well as genes typically expressed in venous ECs, *PLVAP* and *POSTN* (Fig. 2c,l and Extended Data Fig. 2g)*. NPR3* expression, which was also detected in pulmonary arterial ECs, suggests a continuum of *NPR3* expression along this axis. SCENIC analysis showed endocardial_ec enriched for *GATA4* and *GATA6* (Extended Data Fig. 3g), encoding cardiogenic transcription factors involved in the morphogenesis of the outflow tract and atrioventricular canal^50,51^. The expression of these transcription factors in the endocardium is relevant to their role in development, but also suggests that they could have a homeostatic function in the adult endocardium.

### Inferring drug targets in arterial, venous and endocardial ECs

We used drug2cell^52^, an in silico drug screening tool leveraging known drug–protein interactions, to predict potential cellular drug targets across different vascular beds. We found P-selectin (encoded by *SELP*), the target of the humanized monoclonal antibody inhibitor crizanlizumab, in aorta, coronary and pulmonary artery ECs, as well as venous and endocardial ECs (Fig. 2k). These data suggest that, although crizanlizumab is used to prevent microvascular occlusions in sickle cell disease^53^, its effect could extend to other vascular beds.

Furthermore, *CHRM3* (encoding muscarinic acetylcholine receptor 3 (M3R)) is the target of M3R agonist pilocarpine, used in glaucoma, ocular hypertension and xerostomia, and of antagonists revefenacin and aclidinium bromide, used in chronic obstructive pulmonary disease treatment. Here we show that kidney, aorta and coronary artery ECs can be cellular targets of these drugs (Fig. 2k). This provides the opportunity to evaluate these drugs as regulators of vascular tone in these blood vessels via modulation of endothelial nitric oxide release^54^. Interestingly, *CHRM3* was implicated in hypertension^55^ and vasoconstriction following endothelial injury^56^, coinciding with observed enrichment in aorta and coronary artery ECs (Fig. 2c).

Triparanol, a DHCR24 inhibitor and cholesterol-lowering medication, was withdrawn owing to multiple side effects^57^. Analysis with drug2cell highlights that DHCR24 inhibition by triparanol might impact littoral cell function (Fig. 2k). Carbimazole and propylthiouracil, two inhibitors of thyroid peroxidase (encoded by *TPO*) used in hyperthyroidism, were also predicted to target littoral cells, as well as spleen_art_ec (Fig. 2k). After absorption, the active form of carbimazole can cause cellular damage to spleen and other organs in rats through increased oxidative stress^58,59^. Furthermore, propylthiouracil was shown to cause splenomegaly^60^, suggesting that these side effects could be mediated by splenic EC dysregulation.

Thus, the definition of angiotypic and organotypic diversity of macrovascular ECs provides insights into cell-specific drug targets, highlights candidates for drug repurposing and links mechanistic inferences to specific cell states.

### Organotypic specializations of microvascular ECs

**Fig. 3:**
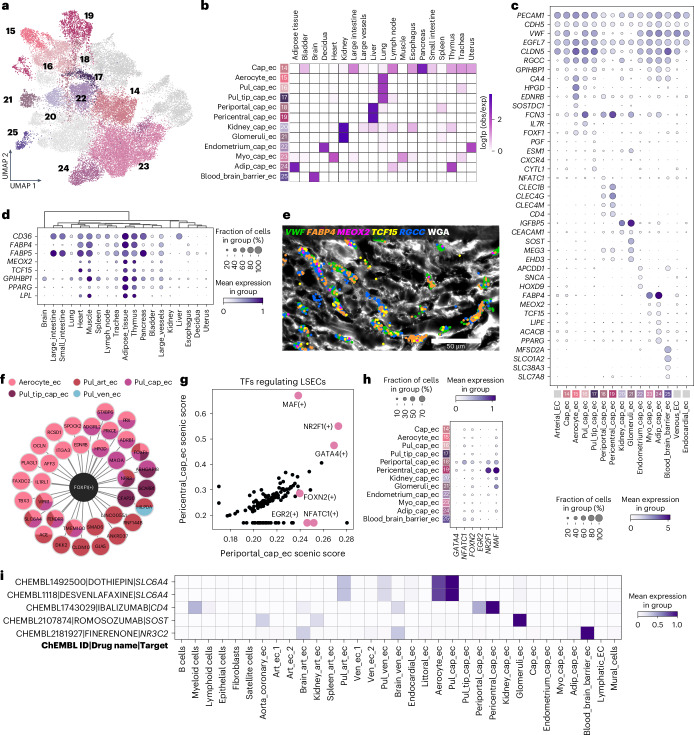
Endothelial heterogeneity within the microvasculature. **a**, UMAP representation of capillary cell states identified across blood ECs from all tissues. **b**, Heatmap representation of cell state enrichment per tissue. **c**, Dot plot representation of signatures of capillary EC states. **d**, Dot plot shows expression of genes encoding proteins involved in fatty acid metabolism within the capillary EC compartment of tissues. **e**, Multiplexed smFISH analysis in cardiac atrial samples showing fatty acid processing related gene markers (*MEOX2*, *FABP4* and *TCF15*) in cardiac capillary ECs (*RGCC*), with EC marker *VWF* and WGA counterstaining for cell membranes. **f**, Downstream targets of lung-specific *FOXF1* (black) across pulmonary EC populations. **g**, Transcription factor enrichment (SCENIC score) of liver periportal (*x* axis) and pericentral (*y* axis) capillary EC populations. **h**, Dot plot shows expression of genes encoding liver pericentral and periportal transcription factors across all capillary EC states. **i**, Matrix plot visualization of inferred capillary EC targets of drugs identified using drug2cell. LSEC, liver sinusoidal endothelial cell; TF, transcription factor.

Capillary ECs were subclustered to reveal 12 states, one of which was shared across most organs (cap_ec), whereas the remainder demonstrated organ specificity (Fig. 3a,b and Supplementary Table 6).

### Fatty acid metabolism in muscle and adipose capillary ECs

**Extended Data Fig. 5:**
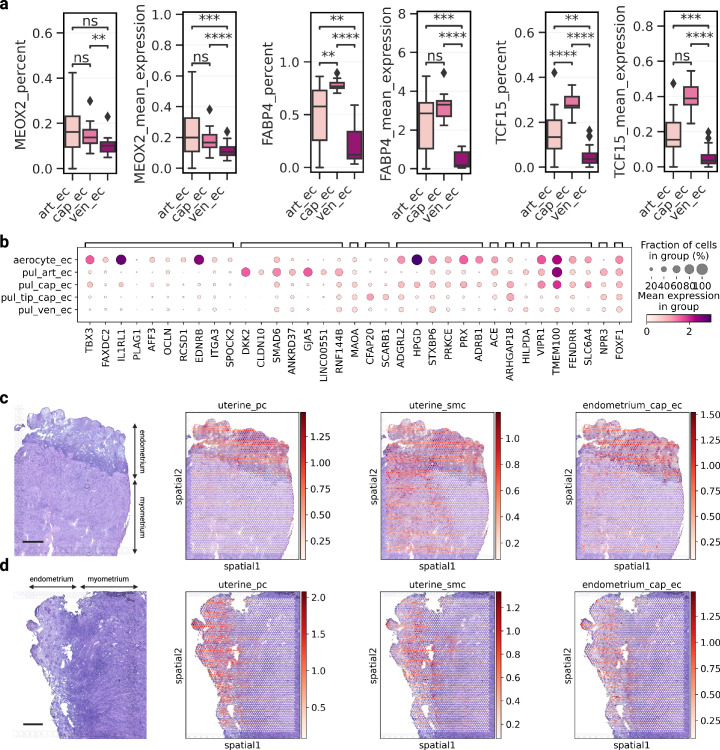
Capillary EC characterisation. **a**, Quantification of fatty acid related gene markers across arterial (art_ec, *GJA5*+/*VWF*+), capillary (cap_ec, *RGCC*+/*VWF*+) and venous (ven_ec, *ACKR1*+/*VWF*+) EC using multiplexed-smFISH. n = 8 sections, 1 donor. Statistical analysis was performed using the Wilcoxon Rank Sum Test with Benjamini-Hochberg adjustment. ns = not significant. ** = adjusted p value <0.01. *** = adjusted p value < 0.001. **** = adjusted p value < 0.0001. For box plots in **a**, the centre line shows the median, the box limits represent the 25th and 75th percentiles, the whiskers show the minimum and maximum values, and the dots represent potential outliers. **b**, Dotplot shows expression of FOXF1 downstream targets genes across lung EC populations. Fraction of expressing cells and average expression within each cell type is indicated by dot size and colour, respectively. **c** & **d**, H&E staining and cell2location predicted cell abundance (5% percentile) of uterine vascular cell states (uterine_pc, uterine_smc, and endometrium_cap_ec) in the endometrium and myometrium location of the uterus in the (**c**) proliferative state, and (**d**) secretory state. Scale bars = 1 mm.

A population restricted to heart and skeletal muscle (myo_cap_ec) expressed genes crucial for fatty acid uptake, suggesting that these contribute to meeting the high energy requirements of muscular tissue. Markers included *FABP4* and *FABP5* (encoding fatty acid binding proteins) and transcription factor genes *MEOX2* and *TCF15* (Fig. 3b–e and Extended Data Fig. 5a), which induce *CD36* (CD36) and *LPL* (lipoprotein lipase) to facilitate fatty acid transport across ECs^17,61^. Furthermore, an adipose tissue–enriched capillary EC population (adip_cap_ec) shared these signatures and expressed lipogenic *ACACB* and adipogenic *PPARG*^62^ (Fig. 3a–d). Adip_cap_ec also comprised cells from the thymus, likely due to thymic adipogenesis during aging^63^.

### Lung microvascular ECs include three cell states

Three lung microvascular EC populations were defined, including aerocytes (aerocyte_ec), pulmonary capillaries (pul_cap_ec) and tip cells (pul_tip_cap_ec) (Fig. 3a–c and Extended Data Fig. 5b). Pul_cap_ec and aerocyte_ec shared expression of epinephrine/norepinephrine responsive marker *ADRB1*, serotonin-related *SLC6A4*, and vasoactive intestinal peptide receptor 1, encoded by *VIPR1* (Extended Data Fig. 1c). Pul_cap_ec signatures overlapped with human lung general capillary and mouse gCap ECs^23,64^, expressing *IL7R* and *FCN3* (Fig. 3c). Additionally, aerocytes, which are ECs at the forefront of alveolar gaseous exchange, expressed *EDNRB*, *CA4*, *SOSTDC1* and prostaglandin-related *HPGD*^23,64^ (Fig. 3c). The expression of prostanoid-related genes in pulmonary microvascular ECs, and in pulmonary arterial and venous ECs, further support that this pathway may contribute to the lower blood pressure within the pulmonary vasculature.

Drug2cell predicted *SLC6A4*-expressing aerocytes and pulmonary capillary ECs as targets of the serotonin/norepinephrine reuptake inhibitors (SNRIs) dothiepin and desvenlafaxine, which are used in antidepressant treatment (Fig. 3i). These findings suggest that targeting of pulmonary microvascular ECs may contribute to respiratory-related morbidity and mortality associated with SNRI use^65^.

SCENIC analysis revealed *FOXF1* (encoding forkhead box F1) as a regulator of all lung EC populations, with shared and cell-state-specific downstream targets (Fig. 3f and Extended Data Fig. 5b), suggesting that the same transcription factor can regulate downstream targets with angiotypic and organotypic specificities. Notably, endothelial FOXF1 inhibits lung fibrosis in mice, and mutations cause congenital alveolar capillary dysplasia with misalignment of pulmonary veins in humans^66,67^.

The third lung-enriched EC population, pul_tip_cap_ec, expresses angiogenic tip cell markers *PGF*, *CXCR4* and *ESM1*, previously described in lung cancer^68^, and *CYTL1*, encoding a pro-angiogenic cytokine produced by endothelial colony-forming cells^69^ (Fig. 3c). This indicates pul_tip_cap_ec as a population with angiogenic potential and/or an endothelial progenitor state.

### Glomerular ECs display a distinctive molecular signature

Kidneys enriched for two microvascular EC populations: glomeruli_ec and kidney_cap_ec (Fig. 3b). Glomerular ECs expressed *EHD3*, encoding an endosomal transport protein involved in fenestrae formation and glomerular filtration^70^, and *MEG3*, implicated in diabetic kidney disease^71^ (Fig. 3c). Glomeruli_ec also expressed sclerostin (encoded by *SOST*) (Fig. 3c), a Wnt inhibitor that reduces bone formation and prevents vascular calcification in mice^72^. Coincidentally, calcifications in the glomeruli are rare^73^, suggesting that glomerular *SOST* could protect against them. Drug2cell predicted glomerular ECs as a target of romosozumab (Fig. 3i), a SOST inhibitor used in the treatment of osteoporosis^74^, although there are no reported renal side effects. Kidney capillary ECs shared expression of *IGFBP5* with glomeruli_ec (Fig. 3c), implicated in vascular inflammation in diabetic kidney disease^75^. Furthermore, kidney_cap_ec showed expression of capillary marker *RGCC*, absent in glomeruli_ec (Fig. 3c), which may represent peritubular and/or vasa recta capillaries as highlighted in mice^27^.

### Zonation of hepatic capillary EC populations

In hepatic lobules, zonation occurs across the portal-central axis in hepatocytes, mesenchymal cells and ECs. We confirm the presence of two liver sinusoidal capillary EC populations, including periportal (periportal_cap_ec) and pericentral (pericentral_cap_ec) ECs^76^. Pericentral capillary ECs enriched genes encoding for C-type lectins (*CLEC1B*, *CLEC4G* and *CLEC4M*) and lectin pathway ficolins (*FCN2* and *FCN3*) (Fig. 3c and Extended Data Fig. 1f), related to the complement cascade and immune regulation^77,78^. Conversely, periportal ECs enriched for markers broadly expressed across vascular beds of other organs (*MGP*, *AQP1* and *CLEC14A*) and the capillary EC marker *RGCC* (Extended Data Fig. 1c).

Based on their expression of *CD4*, liver sinusoidal ECs were identified by drug2cell as a putative target of the anti-CD4 monoclonal antibody ibalizumab (Fig. 3c,i). This is used to prevent HIV entry into CD4^+^ T cells^79^; however, the role of CD4 and the effect of ibalizumab on pericentral and periportal ECs remains unexplored. SCENIC revealed genes encoding transcription factors regulating liver capillary ECs, including *FOXN2*, *EGR2* and *NFATC1*, with *MAF*, *NR2F1* and *GATA4* scoring more highly in pericentral ECs (Fig. 3g,h).

### The uterus contains endometrium-specific capillary ECs

We identified a novel population derived from decidualized and non-decidualized uterine tissue (endometrium_cap_ec) expressing *APCDD1* and *SNCA*, encoding α-synuclein, which regulates the release of molecules in endothelial Weibel–Palade bodies^80^, as well as *HOXD9* (Fig. 3a–c). Localization of these ECs within the endometrium was confirmed using uterus spatial transcriptomics data (Extended Data Fig. 5c,d).

### Brain capillary ECs show features of the blood–brain barrier

We defined a brain-enriched capillary cluster (blood_brain_barrier_ec) that expressed genes encoding known markers of blood–brain barrier ECs, including solute carriers *SLCO1A2*, *SLC38A3* and *SLC7A8* and the fatty acid transporter and transcytosis regulator *MFSD2A*^81–85^ (Fig. 3a–c). Drug2cell predicted blood_brain_barrier_ec as a target of finerenone, an antagonist of the mineralocorticoid receptor (encoded by *NR3C2*) (Fig. 3i). This drug reduces pro-inflammatory and pro-fibrotic processes in chronic kidney disease^86,87^, and, although it does not cross the blood–brain barrier, it may target mineralocorticoid receptors within these cells^86,87^.

In summary, we demonstrate that organotypic microvascular ECs display transcriptional signatures related to tissue function. Notably, contrasting what was reported in mice^17^, in humans, *VWF*, a key regulator of coagulation, was detected almost ubiquitously in ECs, except for aerocytes, kidney and liver capillary ECs (Figs. 2c and 3c).

### VSMCs and pericytes demonstrate angiotypic signatures

**Fig. 4:**
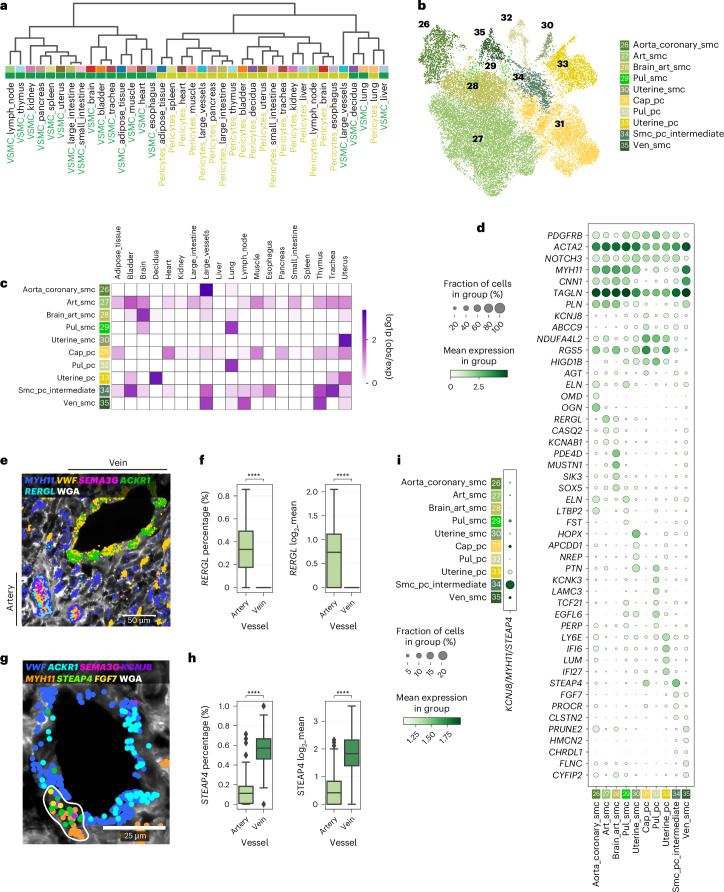
Pericyte and SMC heterogeneity across the human vascular bed. **a**, Dendrogram of hierarchical clustering of mural cell populations subset per organ. Top color bar: organ. Bottom color bar: mural subtype. **b**, UMAP representation of mural cell states identified across all tissues. **c**, Heatmap representation of cell state enrichment per tissue. **d**, Dot plot representation of transcriptional signatures of VSMC and pericyte cell states. **e**, Multiplexed smFISH analysis showing *RERGL* expression in arterial VSMCs (*MYH11*^+^). Expression of EC marker *VWF*, arterial and venous EC markers *SEMA3G* and *ACKR1,* respectively, are shown. WGA was used for cell membrane counterstain. **f**, Box plot representation of *RERGL* expression within arteries and veins across all ROIs captured using multiplexed smFISH. Left, percentage of bins per arterial or venous VSMC cluster expressing *RERGL*. Right, mean expression of *RERGL* in arterial and venous VSMCs (expressing bins only). *n* = 297 vessels, 8 sections, 1 donor. Statistical analysis was performed using the Wilcoxon rank-sum test with Benjamini–Hochberg adjustment. **** adjusted *P* < 0.0001. **g**, Multiplexed smFISH analysis of smc_pc_intermediate markers *STEAP4* and *FGF7* co-expressed with VSMC marker *MYH11* and pericyte marker *KCNJ8* around an *ACKR1*^+^ vein. Absence of arterial EC marker *SEMA3G* confirms venous identity. WGA was used for cell membrane counterstain. **h**, Box plot representation of *STEAP4* expression within arterial and venous VSMCs across all ROIs captured using multiplexed smFISH. Left, percentage of bins per arterial or venous VSMC cluster expressing *STEAP4*. Right, mean expression of *STEAP4* in arteries and veins (expressing bins only). *n* = 297 vessels, 8 sections, 1 donor. Statistical analysis was performed using the Wilcoxon rank-sum test with Benjamini–Hochberg adjustment. **** adjusted *P* < 0.0001. For box plots in **f** and **h**, the center line shows the median; the box limits represent the 25th and 75th percentiles; the whiskers show the minimum and maximum values; and the dots represent potential outliers. **i**, Dot plot representation of *STEAP4* co-expression with VSMC marker *MYH11* and pericyte marker *KCNJ8*.

VSMCs form the muscular layer of arteries and veins, with arterial walls being thicker to withstand higher blood pressures. Furthermore, pericytes form a discontinuous layer surrounding capillaries, providing crucial homeostatic and functional support for the microvasculature^4^. Hierarchical clustering of pericytes and VSMCs across organs largely demonstrated transcriptional similarity within these two cellular compartments (Fig. 4a), suggesting that, like ECs, mural cell specialization occurs predominantly by angiotypic cues.

By subsetting the mural cells, we identified 10 VSMC and pericyte populations (Fig. 4b–d and Supplementary Table 6). An artery-specific VSMC population (art_smc) was characterized by expression of *RERGL*, encoding a tumor suppressor^88^, *CASQ2* (calsequestrin 2), associated with calcium storage and potentially regulating vasoconstriction^89^, and potassium channel *KCNAB1* (Fig. 4d). Multiplexed smFISH confirmed *RERGL* expression within arterial VSMCs and its absence in venous VSMCs (Fig. 4e,f). Notably, aorta and coronary artery VSMCs (aorta_coronary_smc) expressed ECM-encoding genes *ELN*, *OGN* and *OMD*, like their endothelial counterparts (Figs. 2c and 4c,d), contributing to wall structure and mechanosensing. Venous VSMCs (ven_smc) enriched for ECM-related *HMCN2*, as shown in mouse^5^, and *FLNC* (filamin C).

Within the pericyte compartment, general capillary pericytes (cap_pc) were shared among organs and were characterized by expression of known markers *ABCC9, KCNJ8* and *RGS5,* and absence of pan-VSMC marker *MYH11* (Fig. 4c,d). These also demonstrated *AGT* (angiotensinogen) expression, a crucial renin–angiotensin system component that regulates blood pressure and is predominantly produced by the liver^90^. These were also present in smc_pc_intemediate, a population that exhibits features of both pericytes and VSMCs (*PDGFRB*, *ACTA2* and *MYH11*) (Fig. 4d), suggesting a phenotypically intermediate state^4^. These also expressed *STEAP4*, encoding a metalloreductase involved in inflammation^91^. Multiplexed smFISH showed that venous *MYH11*^+^ cells had higher *STEAP4* expression compared to arterial VSMCs, suggesting a greater presence around veins (Fig. 4g,h). Furthermore, single-cell RNA sequencing (scRNA-seq) co-expression analysis revealed that approximately 20% of *STEAP4*^*+*^ cells co-express both *MYH11* and *KCNJ8* in smc_pc_intermediate cells (Fig. 4i), aligning with previous indications that mural cells constitute a continuum across the vascular axis^4^. Additional markers included coagulation-related protein C receptor (encoded by *PROCR*); *FGF7*, previously identified in lung immune-recruiting pericytes^24^; and *CHRDL1* (encoding chordin-like 1) (Fig. 4d,g), reported to promote angiogenesis under hypoxia, which was also expressed in ven_smc^92^.

### The lung, uterus and brain comprise organotypic mural cells

Our integrated analysis shows that signatures of previously described pulmonary VSMCs (pul_smc) and pulmonary pericytes (pul_pc) are specific to the lung (Fig. 4b–d). In addition to confirming their own expression profiles^24^, we also identified shared features, including expression of *TCF21*, encoding a transcription factor involved in lung development^93^, *PERP* and *EGFL6*. The decidua and uterus also comprised unique pericyte and VSMC populations (uterine_pc and uterine_smc, respectively) (Fig. 4b,c). Uterine_pc expressed interferon signaling markers (*IFI6* and *IFI27*), and *LUM* (lumican) (Fig. 4d), which has known roles in tissue remodeling and potentially contributing to tissue turnover in the menstrual cycle and pregnancy^94^. Similarly to uterine capillary EC, uterine_smc expressed the Wnt inhibitor encoding gene *APCDD1*, involved in vascular remodeling^95^, as well as *HOPX* and *NREP* (Figs. 3c and 4d).

Arterial VSMCs from brain (brain_art_smc) expressed arterial VSMC markers, as well as phosphodiesterase-4D (encoded by *PDE4D*), which mediates VSMC contraction via angiotensin-2 (ref. ^96^) and salt-inducible kinase 3 (encoded by *SIK3*), which regulates VSMC proliferation^97^ (Fig. 4c,d). Thus, our findings demonstrate heterogeneity among mural cells, with angiotypic VSMC signatures that vary between arteries and veins and distinct organotypic populations in lung, brain, uterus and decidua.

### Vascular cell states are reproducible in single-nuclei data

**Extended Data Fig. 6:**
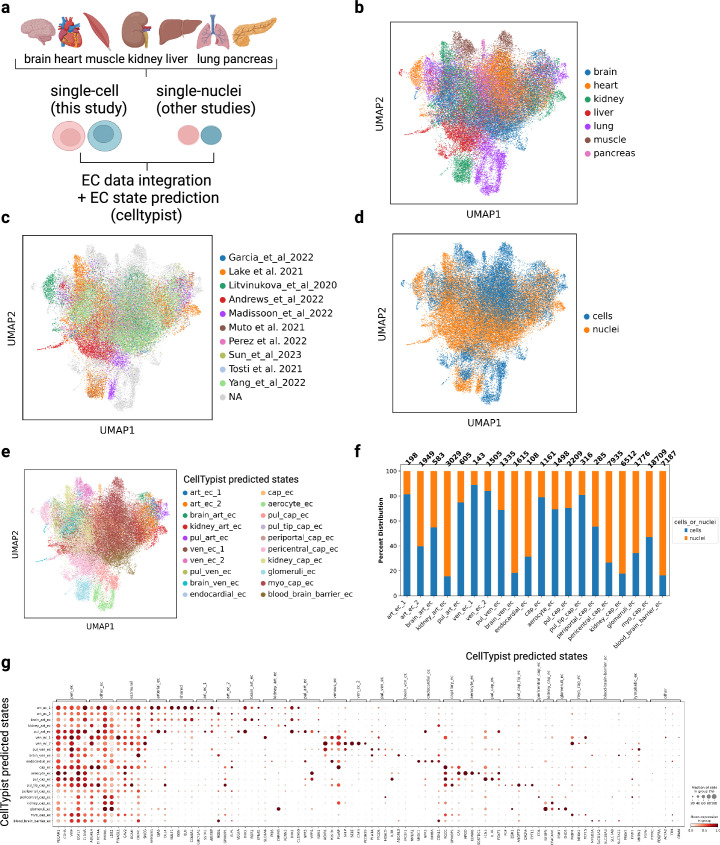
Prediction of EC cell states in single-nuclei datasets. **a**, Schematic diagram of the integration of endothelial cells (ECs) from this single-cell study and published single-nuclei studies from corresponding organs. **b - e**, UMAP representation of (**b**) organs, (**c**) nuclei dataset source, (**d**) suspension type, and (**e**) CellTypist predicted states. **f**, Percentage proportion of EC state predictions from cells or nuclei. **g**, Dotplot of marker gene expression in predicted ECs. Fraction of expressing cells average expression within each cell type is indicated by dot size and colour, respectively. The illustration in (**a**) was created with BioRender.com.

**Extended Data Fig. 7:**
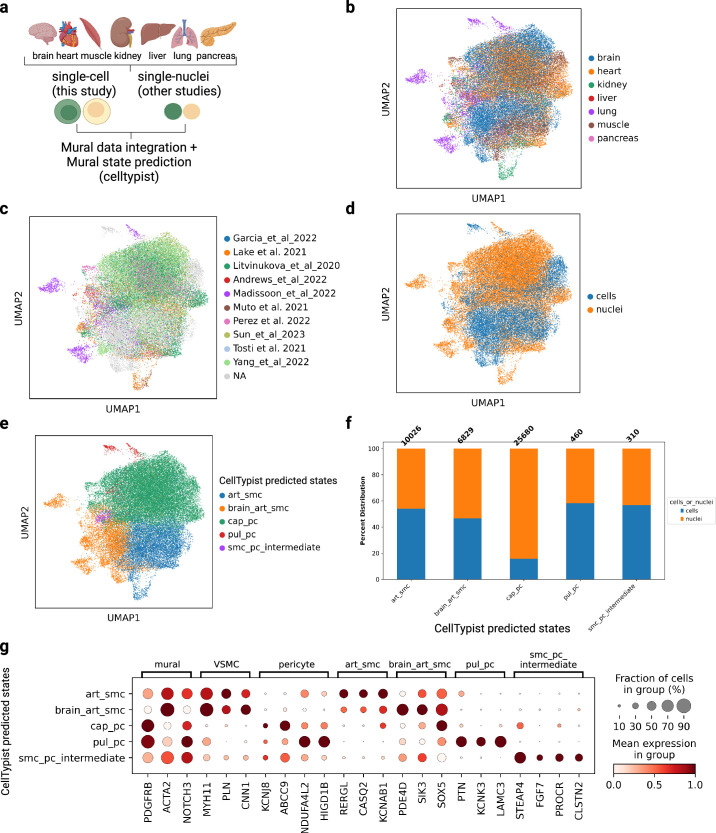
Prediction of mural cell states in single- nuclei datasets. **a**, Schematic diagram of the integration of mural cells from this single-cell study and published single-nuclei studies from corresponding organs. **b - e**, UMAP representation of integrated (**b**) organs, (**c**) nuclei dataset source, (**d**) suspension type, and (**e**) CellTypist predicted states. **f**, Percentage of mural cell state predictions from cells or nuclei. **g**, Dotplot of marker gene expression in predicted mural cell states. Fraction of expressing cells and average expression within each cell type is indicated by dot size and colour, respectively. The illustration in (**a**) was created with BioRender.com.

To test whether vascular cell states are reproduced using additional datasets, we integrated publicly available single-nuclei data from seven organs/tissues with single-cell datasets (Supplementary Table 8). Automated annotation (CellTypist) of vascular cell states demonstrated contribution of both cells and nuclei to the predicted states, with confirmation of relevant marker expression (Extended Data Figs. 6 and 7).

### Angiotypic and organotypic EC–mural cell signaling

**Extended Data Fig. 8:**
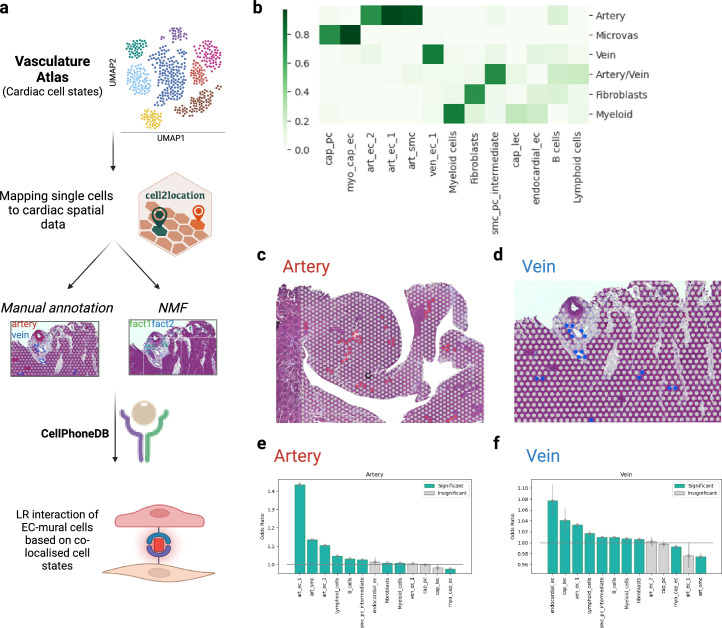
Spatial validation of interacting cell pairs in the heart. **a**, Approach to obtaining relevant cell-cell interactions using non-negative matrix factorisation (NMF) coupled with manual annotation of spatial transcriptomic data. **b**, NMF analysis demonstrating cellular microenvironments. Colour density = loading of the corresponding cell type for the specific NMF factor. **c** & **d**, Example of manual annotation of arteries (**c**) and veins (**d**) using heart spatial transcriptomics data. **e** & **f**, Odds ratio analysis of enriched cell types within manually annotated arteries (**e**) and veins (**f**). Significant cell type enrichment is highlighted in green, with p values < 0.05. Error bars in (**e**) and (**f**) indicate the 10% confidence interval. The illustration in (**a**) was created with BioRender.com.

We used spatial transcriptomics from cardiac tissue to map likely interacting vascular cell partners (Extended Data Fig. 8a). This confirmed expected cell–cell co-localization, such as arterial ECs with arterial VSMCs, capillary ECs with pericytes and venous ec_1 within the venous compartment, with smc_pc_intermediate in both artery and vein niches (Extended Data Fig. 8b). Furthermore, odds ratio analysis of manually annotated regions confirmed these results (Extended Data Fig. 8c–f). Endocardial_ec were also enriched in veins, likely owing to their partially overlapping transcriptional profiles with venous ECs.

**Extended Data Fig. 9:**
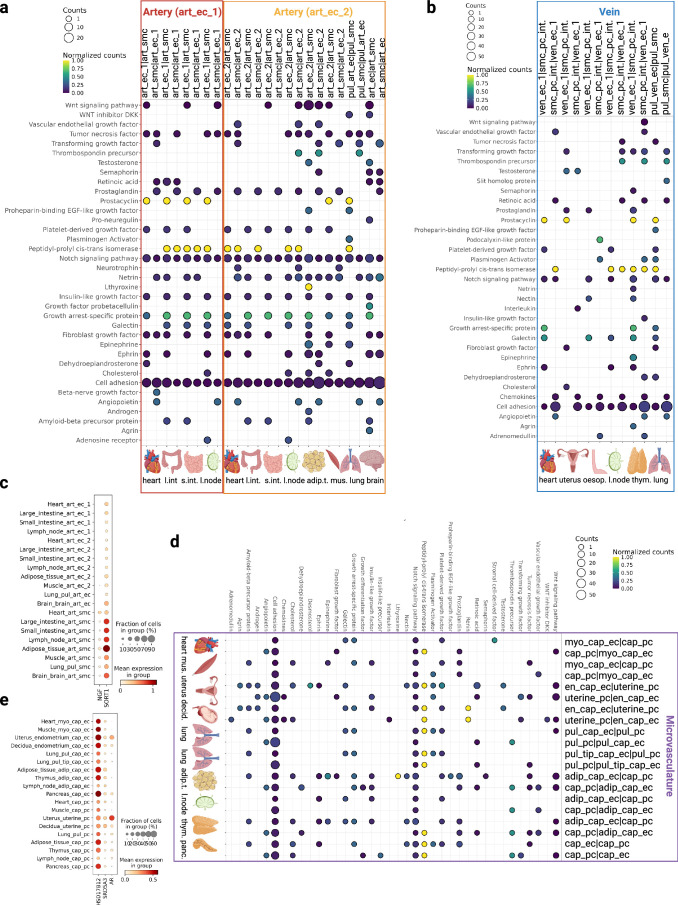
Organotypic intercellular signalling between endothelial and mural cells. **a** & **b**, Ligand-receptor (LR) pairs grouped by pathways in arteries (**a**) and veins (**b**). **c**, Dotplot shows expression of genes encoding factors involved in beta nerve growth factor in arterial microenvironments. Fraction of expressing cells and average expression within each cell type is indicated by dot size and colour, respectively. **d**, LR pairs grouped by pathways in microvasculature. Dot size is the absolute number of LR pairs in a pathway and the colour is the normalised number of LR pairs within a pathway. **e**, Dotplot shows expression of genes encoding factors involved in androgen signalling in capillary microenvironments. Fraction of expressing cells and average expression within each cell type is indicated by dot size and colour, respectively. l.int = large intestine, s.int = small intestine, l. node = lymph node, adip.t = adipose tissue, mus. = muscle, oesop. = oesophagus, decid. = decidua, thym. = thymus, panc. = pancreas. The illustrations in (**a**), (**b**) and (**d**) were created with BioRender.com.

CellPhoneDB^98^ was used to predict interacting cells based on their expression of ligand–receptor (LR) pairs. LR interactions were predicted between ECs and mural cells within arterial, venous and capillary niches across organs (Extended Data Fig. 9 and Supplementary Table 10).

The Notch pathway, a key player in vessel formation and stabilization^99^, involves interactions between Jagged (JAG) or Delta-like family (Dll) ligands with Notch transmembrane receptors on neighboring cells. Specific NOTCH LR interactions exhibit vessel-specific patterns, such as NOTCH2-dependent signaling being enriched in arterial VSMCs, whereas NOTCH3 is present across all vessels with some organ specificity, such as lack of NOTCH3-dependent interactions in the uterus veins (Supplementary Data Fig. 7a). Vice versa, NOTCH1-dependent and NOTCH4-dependent signaling is predominant in arterial ECs. Interestingly, NOTCH signaling showed some organ specificity in veins and microvascular endothelium. Interactions such as JAG1/2–vasorin (*VASN*) could regulate human vascular function, as suggested by the role of vasorin in VSMCs and blood pressure regulation in mice^100^. Fibronectin-mediated interactions were uniformly enriched in arteries across organs, whereas they showed some tissue specificity in veins and capillaries. Conversely, collagen-dependent interactions were largely restricted to veins and capillaries (Supplementary Data Figs. 7b and 8), reflecting differences in vascular basement membrane composition.

**Fig. 5:**
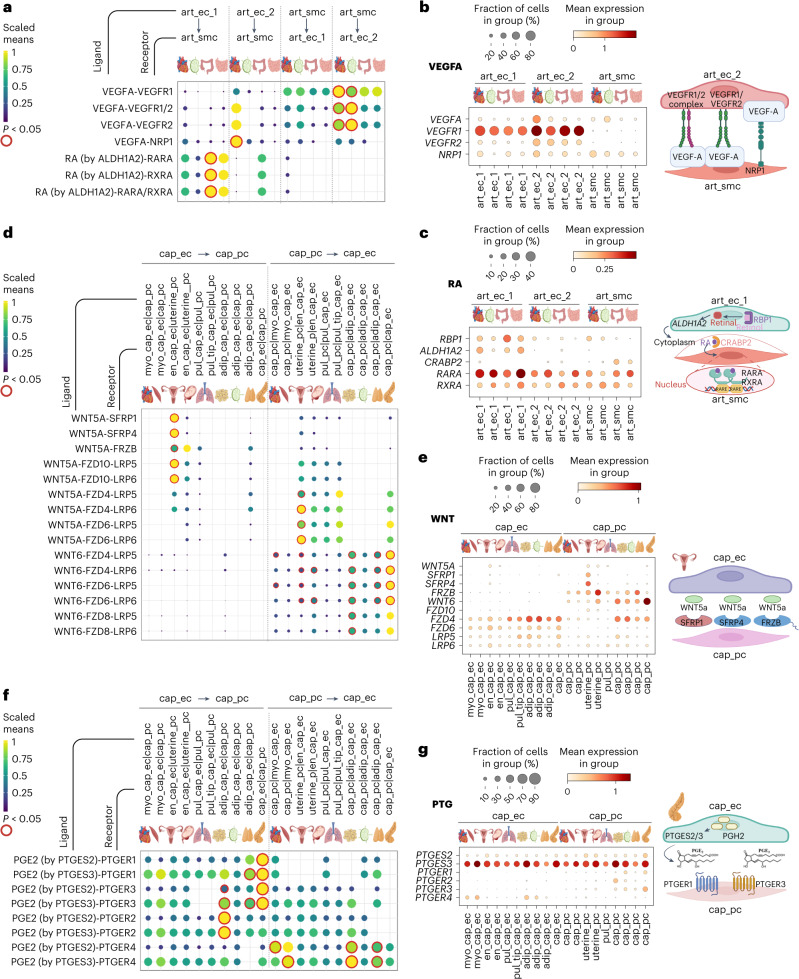
Organ-specific signaling between ECs and mural cells. **a**, LR interactions in VEGFA and RA signaling between ECs and mural cells in arteries. The *y* axis shows specific LR pairs. Dot size and color represent scaled average gene expression, with red circles indicating significant interactions (*P* < 0.05) as calculated using CellPhoneDB. **b**,**c**, Dot plot showing gene expression of LRs involved in the VEGFA (**b**) and RA (**c**) pathway, and schematics summarizing cell–cell interactions. **d**, LR interactions in the WNT pathway between ECs and mural cells in microvasculature. *y* axis shows specific LR pairs. Dot size and color represent scaled average gene expression, with red circles for significant interactions (*P* < 0.05) as calculated using CellPhoneDB. **e**, Dot plot showing gene expression of LRs involved in WNT signaling and schematic summarizing cell–cell interactions. **f**, LR interactions in the prostaglandin (PTG) signaling pathway between ECs and mural cells in microvasculature. The *y* axis shows specific LR pairs. Dot size and color represent scaled average gene expression, with red circles for significant interactions (*P* < 0.05) as calculated using CellPhoneDB. PTGES, prostaglandin E synthase. **g**, Dot plot of LR gene expression and schematic of PTG intercellular signaling. PGH2, prostaglandin H2. The illustrations in **a**–**g** were created with BioRender.

Angiotypic LR interactions included VEGFA–VEGFR1 and/or VEGFR2 signaling from art_smc to art_ec_2 in heart and lymph node (Fig. 5a,b), suggesting a role in maintaining arterial homeostasis by regulating EC survival and vasodilation^101,102^. Additionally, VEGFA–NRP1 signaling from art_ec_2 to art_smc was enriched in cardiac arteries, which, in mice, is essential for vessel development and VSMC function^103^. Furthermore, retinoic acid (RA) signaling was predicted from arterial endothelium (art_ec_1) to VSMCs in the large intestine (Fig. 5a,c), suggesting that RA has an organotypic function in maintaining homeostasis of intestinal arteries beyond its role in vascular development^104^.

Organotypic interactions included beta nerve growth factor signaling from cardiac VSMCs to ECs (*NGF*–*SORT1*) in arteries (Extended Data Fig. 9a,c), shown to be involved in arterial remodeling^105^. *AR* (androgen receptor) signaling was predicted in the decidualized and non-decidualized endometrial microvasculature, where cells specifically express *AR* and testosterone production enzymes (encoded by *SRD5A3* and *HSD17B12*) (Extended Data Fig. 9d,e). In the human uterus, androgens play a role in health by regulating endometrial proliferation and competence during the establishment of pregnancy and tissue repair during menstruation. Conversely, androgen dysregulation contributes to endometriosis, cancer and infertility^106^. Thus, targeted hormonal-based intervention via endometrium microvasculature might be important for restoring normal vascular function.

Additionally, WNT5A-dependent signaling shows enrichment in endometrial capillaries of the uterus, with specific receptor combinations being expressed on ECs and pericytes (Fig. 5d,e). Given the known role of WNT5A in tissue repair^107^, we speculate that this signaling contributes to tissue remodeling in the uterus during the menstrual cycle. In contrast to WNT5A, WNT6 signaling was exclusive from pericytes to endothelium and more widespread across multiple tissues.

Prostaglandins are lipid-derived signaling molecules that play an important role in pain and inflammation. Here we describe prostaglandin-dependent cellular communications with organotypic differences. By mapping prostaglandin E2 (PGE2) synthases and corresponding prostaglandin-E receptor genes (*PTGER1* and *PTGER3*), we identified an enrichment of this signaling in pancreatic capillary endothelium to pericytes. *PTGER4* signaling was, instead, enriched in muscle, adipose tissue and thymus capillary ECs (Fig. 5f,g).

Overall, microvascular LR interactions demonstrated angiotypic and organotypic differences, suggestive of adaptations to the microenvironment.

## Discussion

Although analysis of specific organs and tissues by scRNA-seq has recently identified the molecular signatures of specialized human vascular cells, the specificity of these signatures across tissues remained unclear. By generating an integrated multi-organ human vascular cell atlas from 19 organs and tissues, we defined 42 vascular populations with shared and tissue-specific features.

One of the challenges with scRNA-seq data integration of multiple organ datasets is appropriate correction of artificial batch effects while preserving biological features. Although there is no accepted standard, multiple computational methods have been developed for complex dataset integration, with scVI being one of the top performers^108^. Using this approach, followed by hierarchical clustering, we showed that transcriptional signatures largely correlated with angiotypic identity (artery versus capillary versus vein), although organotypic specificities exist in line with specialized functions of ECs in certain organs^1,109^, suggesting that developmental origin and tissue function may co-adapt with vascular phenotypes. This was in contrast to previous reports of organotypic specificities of vascular cells in a mouse study where ambient RNA removal pipelines and integration were not reported^17^. Nevertheless, to help reveal finer organ-specific gene signatures, future studies would benefit from incorporating batch covariate modeling for technical and biological effects^110^, using tools such as MAST^111^.

Although scRNA-seq has revolutionized vascular research, its sensitivity remains a challenge, whereby the absence of gene detection does not necessarily mean the lack of gene expression. Tissue starting material and dissociation protocols may impact RNA expression and reproducibility of certain cell types^112^. Thus, expanding this vascular atlas with more organs and tissues from additional individuals obtained with complementary single-cell and single-nucleus RNA-seq technologies will further refine its accuracy and comprehensiveness. This will aid future iterations of a human vascular atlas to include analysis of covariates such as age, sex, ancestral and geographic diversity.

The identification of shared and specific molecular signatures across multiple vascular beds can inform broad and localized vascular cellular drug targets. For example, in healthy tissues, we predicted targeting of vascular ECs, other than the microvasculature, by the P-selectin (*SELP*) monoclonal antibody inhibitor crizanlizumab. However, P-selectin is upregulated on capillary ECs during sickle cell crisis^113^ when the drug is needed. Thus, integrating healthy and disease datasets is required to reveal shifts in gene expression and pinpoint vascular cell targets relevant to the disease of interest. This will offer mechanistic insights into drug targets and off-target effects for preclinical screening and potential therapeutic opportunities.

To prioritize functional validation, the development and implementation of high-resolution and multi-modal spatial approaches will enable correlation of gene and protein expression with (sub)cellular localization, which will open up insights into the vasculature in health and disease. In addition, the development of emerging human/humanized in vitro and in vivo systems will allow loss-of-function and gain-of-function studies and drug screening in vascular cells.

In summary, our study provides a unique open-access resource for understanding the molecular identities and adaptations of ECs and mural cells in different vascular beds and organs, provided at https://www.vascularcellatlas.org/. Crucially, this multi-tissue healthy vascular cell atlas can be further used as a reference to understand the contribution of vascular cells to disease and guide strategies for developing targeted therapeutics for specific vascular beds across organs.

## Methods

### Research ethics for donor tissues

All tissue samples were obtained from transplant donors after research ethics committee approval and written informed consent from donor families. Human heart samples used for Molecular Cartography and Visium experiments were acquired by Imperial College London under agreement REC reference 16/LO/1568, London, London Bridge Research Ethics Committee. Human ileum, lymph node, spleen and skeletal muscle tissues were obtained from the Cambridge Biorepository for Translational Medicine under agreement REC reference 15/EE/0152, East of England Cambridge South Research Ethics Committee, and approved by the National Institute for Health and Care Research (NIHR203312).

### Data sources

#### De novo generated single-cell data

Donor information for spleen and lymph node tissue used for scRNA-seq is provided in Supplementary Table 2. Upon removal, spleen or lymph node samples were transferred to HypoThermosol (Sigma-Aldrich, H4416-100ML) and shipped to the Wellcome Sanger Institute on ice within 24 h. Samples were washed in DBPS, cleaned from fat and connective tissue, minced and digested in the mixture of Liberase TH (Roche, 05401135001) and DNAse-I (Roche, 4716728001) in RPMI media. After 30 min, digestion was stopped with 10% of FBS in RPMI, and digested cells were strained through a 70-μm strainer and pelleted at 500*g* and underwent red blood cell lysis using a buffer from eBioscience (00-4333-57). If there was remaining tissue after 30 min, it was digested with a fresh mixture of Liberase TH and DNAse-I in RPMI for an additional 15–30 min and underwent red blood cell lysis. To isolate stromal cells, either magnetic (donor A51) or FACS (donors A59, A60 and A61) sorting was employed. Magnetic sorting to deplete CD45^+^ cells was performed using a magnetic sorting kit from Miltenyi Biotec including LS Columns (130-042-401) and CD45 MicroBeads, human (130-045-801). For FACS sorting, cells were resuspended in the FACS buffer (0.5% FBS and 2 mM EDTA in PBS), underwent blocking in TruStain FcX (BioLegend, 422302) for 10 min and were stained with a mixture of CD31 (anti-CD31 FITC, WM59 clone; BD Biosciences, 555445; 1:50 dilution), CD45 (CD45 BV785, HI30 clone (mouse); BioLegend, 304048; 1:50 dilution), PDPN (PE anti-human PDPN, NC-08 clone; BioLegend, 337003; 1:50 dilution) and THY1 (APC anti-human CD90(THY1), 5E10 clone (mouse); BD Biosciences, 561971; 1:50 dilution) antibodies and DAPI for 30 min. After staining, cells were washed and analyzed using Sony SH800 or Sony MA900 sorters (Cell Sorter Software version 3.1.1) with a 130-μm nozzle. Two cell fractions—one, positive for CD31^+^ and THY1^+^, and the other, positive for THY1^+^—were sorted into the 1% FBS in PBS solution after standard QC, including debris and dead cell removal. Both total and sorted fractions were resuspended to recommended concentration (10^6^ per milliliter) and loaded on a 10x Genomics chromium controller to generate emulsion of cells in droplets using a Chromium Next GEM Single Cell 5′ Kit v2 (no. 1000263). GEX libraries were prepared using a Library Construction Kit (no. 1000190), and sequencing was performed with a NovaSeq 6000 sequencer (Illumina).

#### Publicly available scRNA-seq data

The list of publicly available scRNA-seq datasets used in this study can be found in Supplementary Table 1 and includes samples from the heart, lung, skeletal muscle, decidua, uterus, small and large intestine, kidney, trachea, brain, esophagus, liver, blood and the Tabula Sapiens cross-tissue study. The metadata of donors (age, sex, organ, library preparation kit and study) are listed in Supplementary Table 2. Repositories where publicly available data are stored can be found in the ‘Data availability’ section.

### scRNA-seq analysis

#### Data pre-processing

Datasets other than Tabula Sapiens data were mapped with CellRanger (version 3.0) and STARsolo (version 2.7.3a) to human genome GRCh38 version 3.0.0, with ambient RNA removed with CellBender (version 0.2.0). Pre-processed Tabula Sapiens data were used^6^. Python (version 3), AnnData (versions 0.8.0 and 0.9.1) Pandas (versions 1.5.2 and 1.5.3), NumPy (versions 1.21.6 and 1.23.5), SciPy (versions 1.8.0 and 1.9.3), Matplotlib (version 3.5.2), Seaborn (version 0.12.2) and Scanpy (versions 1.8.2 and 1.9.1) were used for QC and downstream processing. Doublets were detected using Scrublet (version 0.2.3)^114^, and cells with a Scrublet score ≥ 0.4 and adjusted Benjamini–Hochberg-corrected *P* ≤ 0.7 were predicted as doublets and filtered out. Low-quality cells were filtered out by the following criteria: minimum number of reads = 500, minimum number of genes = 300, percentage of mitochondrial genes ≤ 0.4, percentage of ribosomal genes ≤ 0.3. Data were then normalized to 10^4^ and log transformed (ln(x + 1)) using the SCANPY workflow^115^. Untransformed reads were stored in layer (‘counts’).

#### Data selection, integration, clustering and annotation

Dimensionality reduction and batch correction of the global cell type dataset (~800,000 cells) were carried out using the scVI model (version 0.9.1)^116^ with untransformed raw counts. In total, 5,000 highly variable genes were selected per dataset_group in the ‘seurat_v3’ flavor. The number of layers in the scVI model was set to 2 while providing the donor as batch key, categorical covariates such as kit and dataset_group and continuous covariate keys such as percentage of ribosomal genes, percentage of mitochondrial genes, number of genes and number of counts. Leiden clustering revealed broad cell types that were annotated based on the expression of known marker genes: endothelial cells (*PECAM1*, *CDH5*, *VWF*), mural cells (*ACTA2*, *MYH11*, *RGS5*), fibroblasts (*DCN*, *PDGFRA*, *SCARA5*), satellite cells (*PAX7*, *DLK1*, *MYF5*), epithelial cells (*EPCAM*, *CLDN7*, *CDH1*), myeloid cells (*C1QA*, *MRC1*, *MARCO*), B cells (*MS4A1*, *CD79A*, *CD19*) and lymphoid cells (*CD3E*, *KLRB1*, *NKG7*).

For downstream analysis of vascular cells, samples were downsampled to a maximum of 1,500 cells per donor per organ, performed in an unbiased manner using the ‘subsample’ function in SCANPY^115^. Each organ was represented by at least two donors, with no single donor contributing more than 85% of the vascular cells for the given organ. Five donors (621B, 637C, A36, D496, D503) providing data from peripheral blood mononuclear cells (PBMCs), were lost between the all-cell object and the vascular object due to a lack of vascular cells within these samples. A total of 67,398 from cells were used for downstream clustering, annotation and analysis. Data integration was carried out for the vascular object, following the same parameters outlined above, except with a selection of 1,500 highly variable genes. Unbiased clustering was performed using the Leiden method, separating cells into four compartments: blood endothelial cells (excluding littoral cells) (*PECAM1*^+^, *PROX1*^−^), littoral cells (*PECAM1*^+^, *CD8A*^+^, *ACKR1*^+^) lymphatic endothelial cells (*PECAM1*^+^, *PROX1*^+^) and mural cells (*ACTA2*^+^, *MYH11*^+^, *ABCC9*^+^).

Data integration was further carried out within each vascular cell compartment using 1,500 highly variable genes and the number of layers = 1. An iterative exploration of hierarchical clustering commenced with a low Leiden resolution of 0.2, followed by unbiased subclustering within each broader cluster (resolution ~ 0.2). New subclusters were assessed for the presence of marker genes by using the Wilcoxon rank-sum test with Benjamini–Hochberg adjustment within the ‘rank_genes_groups’ function in SCANPY^115^. Subtypes were classified based on their possession of distinct marker genes and/or their substantial association (≥80% fraction) with specific organs, whereas cell states consistently present across organs were classified as shared. For visualization, data were reintegrated using scVI in objects where clusters were removed. Cell state enrichment (log1p obs/exp) heatmaps were generated using tissues contributing more than 10 cells per state.

#### Differential gene expression analysis

Differential gene expression analysis between cell types and states was performed using Wilcoxon rank-sum test with Benjamini–Hochberg adjustment on all standard size-normalized genes, filtered for adjusted *P* < 0.05 and |log_2_ fold change (FC)| > 0.5, using the SCANPY ‘rank_genes_groups’ function. Marker genes displayed in figures were manually selected based on knowledge from existing literature and/or high expression and specificity within cell types and states.

#### Gene regulatory network analysis

The SCENIC pipeline^43^ (version 0.11.2) was used to predict transcription factors that regulate different types of vascular cells. Gene regulatory network (GRN) analyses were performed on the high-resolution vascular cell annotations, once across all vascular cell types and once across the subset of capillary EC/pulmonary vascular cell types. First, gene regulatory interactions were calculated based on the co-expression, which was further pruned using known transcription factor binding motifs, and then compartment-specific regulatory modules (regulons) were constructed. AUCell was used to score the regulons in each cell. For the capillary EC analysis, regulating transcription factors were further selected by being differentially expressed in the cell type of interest (‘scanpy.tl.rank_genes_groups’ implementing Wilcoxon rank-sum test, *P* ≤ 0.05, logFC ≥ 1, fraction expressing ≥ 0.25) compared to all other cell types within the compartment. Similarly, target genes were filtered for genes differentially expressed across compartments (same criteria as transcription factor selection except for logFC ≥ 2). A network of regulatory transcription factors and target genes was then constructed using a customized script.

#### Trajectory inference analysis

scFates^117^ was used to infer zonation patterns of arterial and capillary ECs by trajectory inference. Arterial ECs (aorta_coronary_ec, art_ec_1 and art_ec_2) and capillary ECs (cap_ec) were subset from the vascular object. Dimensionality reduction and batch correction were performed using Harmony^118^ on all highly variable genes, correcting for donors. The principal curve was inferred on uniform manifold approximation and projection (UMAP) with 100 dimensions and five nodes. Pseudotime (zonation) was calculated from the root, and linearity deviation was determined between the start and end milestone, with important features along the trajectory calculated and mapped (UMAP or heatmap representation).

#### Drug2cell

Drug2cell analysis was performed on the global object comprising both vascular cells state annotations and other compartments (Fig. 1b) using the ‘drug2cell’ Python package (version 0.1.0)^52^. The lists of drugs and their corresponding targeting genes were obtained from ChEMBL (version 30) (filtered down to 2,395)^52^. Drug2cell scores in each single cell were computed based on the average expression of the target gene(s). Subsequently, Wilcoxon rank-sum test with Benjamini–Hochberg adjustment was carried out to assess significance of drugs targeting across vascular cell types/states. To further filter on the outcome, the targeting genes were required to be expressed in at least 25% of the cells within the type of interest, with a logFC higher than 2 compared to all other cells. Drugs displayed in figure panels were curated based on these filters, in addition to relevance to the literature. Drug2cell scoring for all vascular cell states and non-vascular cell types can be found in Supplementary Table 9.

#### Spatially resolved cell–cell interaction analysis

Cell–cell interaction analysis was performed on the identified spatial niches (artery, vein and microvasculature) and between the respective enriched cell–cell pairs within these niches using CellPhoneDB (version 4.0.0)^98^ and database version 4.1.0 (https://pypi.org/project/CellphoneDB/) using the statistical analysis method and a microenvironment file to restrict the cell pairs within each organ. Before running the cell–cell interactions analysis, we applied a filter of a minimum of 20 ECs and mural cells per organ. Thus, the interaction analysis is not exhaustive, due to limitations such as capturing enough cells per organ in this current integration analysis. For CellPhoneDB analysis, the significant LR pairs were retrieved based on the following criteria: (1) each of the genes of LR pairs was expressed in at least 10% of the cells in the cell states; and (2) LR complexes specific to two cell states were inferred by the statistical method framework in CellPhoneDB (‘statistical_analysis’, *P* value threshold = 0.05). LR pairs were manually curated into specific signaling pathways as shown in Troule et al.^119^. The Python ktplotspy library^119^ was used to visualize the CellPhoneDB analysis results.

#### Integration of single-cell and single-nuclei data for cross-modality profiling of cell states

Single-nuclei data were obtained from publicly available datasets from seven tissues, including heart, lung, liver, pancreas, brain, kidney and skeletal muscle (Supplementary Table 8).

First, a single-nuclei object from these tissues was obtained across all cell types. Similarly to the all-cell integration approach described above, the dimensionality reduction and batch correction of the global cell type dataset (~970,000 cells) were carried out using the scVI model with untransformed raw counts. In total, 5,000 highly variable genes were selected per study in the ‘seurat_v3’ flavor. The number of layers in the scVI model was set to 3 while providing the donor as batch key, categorical covariates including kit and continuous covariate keys including percentage of ribosomal genes, percentage of mitochondrial genes, number of genes and number of counts. Broad cell types were annotated based on the expression of known marker genes: endothelial cells (*PECAM1*, *CDH5*, *VWF*), mural cells (*ACTA2*, *MYH11*, *RGS5*), fibroblasts (*DCN*, *PDGFRA*, *SCARA5*), epithelial cells (*EPCAM*, *CLDN7*, *CDH1*), myeloid cells (*C1QA*, *MRC1*, *MARCO*), B cells (*MS4A1*, *CD79A*, *CD19*) and lymphoid cells (*CD3E*, *KLRB1*, *NKG7*).

For downstream vascular analysis, each organ was subset to 20,000 cells in an unbiased manner using the ‘subsample’ function in SCANPY. Doublets were removed by maintaining cells with a Scrublet score ≤ 0.4. A total of 67,130 cells were used for downstream clustering, annotation and analysis. Data integration was carried out for the vascular object following the same parameters outlined above, except with the number of layers set to 2. Unbiased clustering was performed using the Leiden method, separating cells into two compartments: blood endothelial cells (*PECAM1*^+^, *VWF*^+^, *CDH5*^+^, *PROX1*^−^) and mural cells (*ACTA2*^+^, *MYH11*^+^, *ABCC9*^+^) (Supplementary Data Fig. 6). Furthermore, single-nuclei data were integrated with single-cell data of the same tissues. Namely, ECs from the single-nuclei dataset were separated and integrated with the ECs from the single-cell dataset. Cell cycle, mitochondrial and ribosomal genes were first removed from the concatenated object. In total, 7,000 highly variable genes were selected per study for either the single-cell or the single-nuclei dataset in the ‘seurat_v3’ flavor. Union of these highly variable genes resulted in 9,720 genes that were further used in scVI integration. The number of layers in the scVI model was set to 1 while providing the donor as batch key, categorical covariate such as kit, study and isolation technique (cell or nuclei) and continuous covariate keys such as percentage of ribosomal genes, percentage of mitochondrial genes and number of genes. The same approach was taken for the mural compartment, using 9,875 genes after union of highly variable genes for scVI integration. Finally, a CellTypist^120^ model was generated based on vascular cell states identified in the seven organ cell dataset using highly variable genes and modeled on the combined single-cell/single-nuclei dataset using best match and majority voting approaches.

### Spatial validation experiments

#### Preparation of Visium spatial transcriptomics slides, library preparation and pre-processing

Freshly isolated human heart samples were fixed in 10% formalin solution for 24 h before transfer to 70% ethanol solution. Samples were then paraffin wax embedded, sectioned at 5-µm thickness and placed on Visium slide capture areas (10x Genomics). Hematoxylin and eosin (H&E) staining was performed according to standard protocols, and imaging was performed using wide-field microscopy (Zeiss Axio Observer). cDNA library preparation was performed according to the manufacturerʼs instructions and sequenced using Illumina NovaSeq 2 × 150 configuration.

Spatial transcriptomics libraries were mapped to the reference genome provided by 10x Genomics (GRCh38 version 3.0.0) using SpaceRanger (version 1.1.0) using default parameters and aligned with sample-relevant H&E images.

#### Visium spatial transcriptomics data analysis

To identify the spatial organisation of the vascular cell types defined by single-cell transcriptomic data analysis in different organs^121^ we applied cell2location (version 0.1.3)^122^ on Visium datasets generated by this study following instructions from cell2location documentation. In brief, we first estimated the reference signature from scRNA-seq/single-nuclei RNA-seq (snRNA-seq) data with a negative binomial regression model. To ensure tissue-specific and robust estimation of the gene signatures, we analyzed only relevant cell types from that organ by excluding cell types with fewer than 10 cells available. Because vascular cells are often a minority in each organ, we included vascular cells also from other organs and included the organ as a batch covariate in the reference mapping step. Additionally, we added the original publication identifier and the 10x experiment type as categorical batch covariates and the number of expressed genes, total unique molecular identifier counts, percentage of mitochondrial gene expression and ribosomal gene expression as continuous batch covariates. We then inferred the cell type abundance in each Visium spot with the learned gene signatures from the first step. For hyperparameters selection, we kept the parameters from the original publications. For heart slides, we used 7 for n_cells_per_location and 20 for alpha. For the uterus slide, we used 8 for n_cells_per_location and 20 for alpha. For Visium slides batch correction, we added the sample identifier as batch. To identify co-localized cell types, we used the non-negative matrix factorization (NMF) algorithm implemented in the cell2location package. Specifically, we pre-filtered for high-quality Visium spots with at least two predicted cell types, each with a minimal abundance of one cell.

To identify the enriched cell type in each manually annotated tissue region—for example, vein and artery—we fitted a univariate regression model with the module statsmodels.formula.api.OLS from the Python package statsmodels version 0.14.0. For the dependent variable, we constructed a binary variable to indicate the membership of each Visium spot in each manual annotation category. For instance, if a spot is manually annotated as artery, then we assign ‘1’ to this spot. For the independent variables, we used the predicted abundance of the cell type from cell2location results. With the Bonferroni method, we corrected the total number of cell types tested for each manually annotated region.

#### RNAscope (smFISH)

Formalin-fixed paraffin-embedded (FFPE) tissues of skeletal muscle, heart and ileum were selected and sectioned at 5-µm thickness onto SuperFrost Plus slides. Slides were incubated at 60 °C for 30 min before staining. Using Leica BOND RX to automate the RNAscope staining on the FFPE sections using an RNAscope LS Multiplex Fluorescent Reagent Kit v2 Assay and an RNAscope LS 4-Plex Ancillary Kit for LS Multiplex Fluorescent (Advanced Cell Diagnostics (ACD) Bio-Techne) as per manufacturer’s instructions, all sections were subjected to heat-induced epitope retrieval enzyme 2 for 15 min at 95 °C and 15 min of protease III before staining protocols were performed. Before running RNAscope probe panels, the RNA quality of the FFPE samples was assessed using multiplex positive (RNAscope LS 2.5 4-plex Positive Control Probe; ACD Bio-Techne, 321808) and negative (RNAscope 4-plex LS Multiplex Negative Control Probe; ACD Bio-Techne, 321838) controls.

The RNAscope list is as follows: RNAscope 2.5 LS Probe - Hs- TINAGL1-C1 (ACD Bio-Techne, 857221-C2), RNAscope 2.5 LS Probe - Hs- EGFL7-C1 (ACD Bio-Techne, 314008), RNAscope 2.5 LS Probe - Hs- LMOD1-C1 (ACD Bio-Techne, 444148), RNAscope 2.5 LS Probe - Hs- MYLK-C3 (ACD Bio-Techne, 533478-C3), RNAscope 2.5 LS Probe - Hs- PROX1-C2 (ACD Bio-Techne, 530248-C2), RNAscope 2.5 LS Probe - Hs- NTS-C4 (ACD Bio-Techne, 512568) and RNAscope 2.5 LS Probe - Hs- LYVE1-C3 (ACD Bio-Techne, 426911-C3).

Some FFPE sections had combined RNAscope and immunofluorescence to visualize the RNA and protein together. Primary antibodies included mouse anti-CDH5 (CD144, IgG1; Thermo Fisher Scientific, 14-1449-82; 1:3,000 dilution) and mouse anti-alpha smooth muscle actin (IgG2a (1A4); Abcam, ab7817; 1:3,000 dilution), in conjugation with secondary antibodies goat anti-mouse (IgG; Thermo Fisher Scientific, A10551; 1:1,000 dilution) and goat anti-mouse (IgG2a; Thermo Fisher Scientific, A1685; 1:1,000 dilution). All nuclei were stained with DAPI (Thermo Fisher Scientific, D1306).

The probes and antibodies were labeled using Opal 520, 570 and 650 fluorophores (Akoya Biosciences, diluted 1:1,000), and one probe channel was labeled using Atto 425-Streptavidin fluorophore (Sigma-Aldrich, diluted 1:500), which was first incubated with TSA-biotin (Akoya Biosciences, diluted 1:400).

Confocal imaging was performed on a PerkinElmer Operetta CLS High Content Analysis System using a ×20 (numerical aperture (NA) 0.16, 0.299 μm per pixel) water immersion objective with a 9–11 z-stacks 2-µm step. Channels: DAPI (excitation (ex.) 355–385 nm, emission (em.) 430–500 nm), Atto 425 (ex. 435–460 nm, em. 470–515 nm), Opal 520 (ex. 460–490 nm, em. 500–550 nm), Opal 570 (ex. 530–560 nm, em. 570–620 nm) and Opal 650 (ex. 615–645 nm, em. 655–760 nm). Non-confocal imaging was performed on a Hamamatsu NanoZoomer S60 fluorescence whole-slide scanner using a ×40 objective lens. Channels: DAPI (ex. 355–385 nm, em. 430–500 nm), FITC (ex. 460–490 nm, em. 500–550 nm), TRITC (ex. 530–560 nm, em. 570–620 nm) and Cy5 (ex. 615–645 nm, em. 655–760 nm). Confocal and non-confocal image stacks were stitched as individual z-stacks using proprietary Acapella scripts provided by PerkinElmer and visualized using OMERO Plus (Glencoe Software).

#### RNAscope quantification

Image quantification was performed using Fiji (version 2.14.0). Background removal for each channel was performed as follows: (1) raw image subtraction with Gaussian blur transformation σ = 50; (2) background subtraction with rolling ball radius of 50 pixels; and (3) reducing pixels with intensity <40 to zero (of an 8-bit image). Three regions of interest (ROIs) were selected for quantification per sample (three donors: 2× ileum, 1× skeletal muscle). ROIs were binned (pixel × pixel) with maximum intensity quantified per channel (ACTA2, CDH5, *TINAGL1*). Bins expressing both ACTA2 and CDH5 were filtered out before determining co-expression of either ACTA2 or CDH5 with *TINAGL1*.

#### Rarecyte CD8a antibody staining

Human spleen FFPE blocks were sectioned using a microtome (Leica, RM2235) at 5-µm thickness and placed on a SuperFrost slide (Thermo Fisher Scientific, 12312148). Slides were dried at 60 °C for 60 min to ensure that tissue sections had adhered to the slides. Tissue sections were deparaffinized and subjected to antigen retrieval using a BioGenex EZ-Retriever system (95 °C for 5 min, followed by 107 °C for 5 min). To remove autofluorescence, slides were bleached with AF Quench Buffer (4.5% H_2_O_2_, 24 mM NaOH in PBS). Slides were quenched for 60 min using the ‘high’ setting with a strong white light exposure, followed by further quenching for 30 min using 365-nm ‘high’ setting using a UV transilluminator. Slides were rinsed with 1× PBS and incubated in 300 µl of Image-iT FX Signal Enhancer (Thermo Fisher Scientific, I36933) for 15 min. Slides were rinsed again, and 300 µl of labeled CD8a primary antibody was added to the tissue, which subsequently was incubated for 120 min in the dark in a humidity tray. CD8a antibody was pre-diluted according to company recommendations. Slides were washed with a surfactant wash buffer, and 300 µl of nuclear staining in goat diluent was added to the slide. Slides were then incubated in the dark for 30 min in a humidity tray. Slides were then washed and placed in 1× PBS. Finally, the slides were coverslipped using ArgoFluor mount media and left in the dark at room temperature overnight to dry. Slides were imaged on the following day using a RareCyte Orion microscope with a ×20 objective, and relevant acquisition settings were applied using Artemis version 4 software.

### Molecular Cartography (multiplexed smFISH)

#### Tissue sections

Freshly dissected right atrial cardiac samples were embedded in OCT-flooded base moulds and frozen in a dry-ice-cooled bath of 2-methylbutane (Sigma-Aldrich, M32631) at −70 °C. Tissues were then sectioned with a cryostat (Leica, CX3050S), and 10-µm-thick sections were placed within the capture areas of cold Resolve Biosciences slides. Samples were then sent to Resolve Biosciences on dry ice to perform Molecular Cartography experiments, as described previously^123^. Upon arrival, tissue sections were thawed and fixed with 4% v/v formaldehyde (Sigma-Aldrich, F8775) in 1× PBS for 20 min at 4 °C. After fixation, sections were washed twice in 1× PBS for 2 min, followed by 1-min washes in 50% ethanol and 70% ethanol at room temperature. Fixed samples were used for Molecular Cartography (100-plex combinatorial smFISH) according to the manufacturer’s instructions (protocol 1.3; available for registered users), starting with the aspiration of ethanol and the addition of buffer DST1, followed by tissue priming and hybridization. In brief, tissues were primed for 30 min at 37 °C, followed by 24-h hybridization of all probes specific for the target genes (see below for probe design details and target list). After the hybridizations step, samples were washed to remove excess probes and fluorescently tagged in a two-step color development process. ROIs were imaged as described below, and fluorescent signals were removed during de-colorization. Color development, imaging and de-colorization were repeated for multiple cycles to build a unique combinatorial code for every target gene that was derived from raw images as described below. Wheat germ agglutinin (WGA) and DAPI were used to counterstain cell membranes and nuclei, respectively.

#### Probe design

The probes for 100 genes were designed using the proprietary design algorithm from Resolve Biosciences. In brief, the probe design was performed at the gene level. For every targeted gene, all full-length protein-coding transcript sequences from the Ensembl database were used as design targets if the isoform had the GENCODE annotation tag ‘basic’^124,125^. The calculation of computationally expensive parts, especially the off-target searches, the selection of probe sequences was not performed randomly but limited to sequences with high success rates. To filter highly repetitive regions, the abundance of *k*-mers was obtained from the background transcriptome using Jellyfish^126^. Every target sequence was scanned once for all *k*-mers, and those regions with rare *k*-mers were preferred as seeds for full probe design. A probe candidate was generated by extending a seed sequence until a certain target stability was reached. A set of simple rules was applied to discard sequences that were found experimentally to cause problems. After these fast screens, every kept probe candidate was mapped to the background transcriptome using ThermonucleotideBLAST^127^, and probes with stable off-target hits were discarded. Specific probes were then scored based on the number of on-target matches (isoforms), which were weighted by their associated APPRIS level^127,128^, favoring principal isoforms over others. A bonus was added if the binding site was inside the protein-coding region. From the pool of accepted probes, the final set was composed by picking the highest-scoring probes. Supplementary Table 7 highlights the gene names and catalog numbers for the specific probes designed by Resolve Biosciences.

#### Imaging

Samples were imaged on a Zeiss Celldiscoverer 7, using the ×50 Plan-Apochromat water immersion objective with an NA of 1.2 and the ×0.5 magnification changer, resulting in a ×25 final magnification. Standard CD7 LED excitation light source, filters and dichroic mirrors were used together with customized emission filters optimized for detecting specific signals. Excitation time per image was 1,000 ms for each channel (DAPI was 20 ms). A z-stack was taken at each region with a distance per z-slice according to the Nyquist–Shannon sampling theorem. The custom CD7 CMOS camera (Zeiss Axiocam Mono 712, 3.45-µm pixel size) was used. For each region, a z-stack per fluorescent color (two colors) was imaged per imaging round. A total of eight imaging rounds were performed for each position, resulting in 16 z-stacks per region. The completely automated imaging process per round (including water immersion generation and precise relocation of regions to image in all three dimensions) was realized by a custom Python script using the scripting API of Zeiss ZEN software (Open Application Development).

#### Spot segmentation

The algorithms for spot segmentation were written in Java and are based on ImageJ library functionalities. Only the iterative closest point algorithm was written in C++ based on the libpointmatcher library (https://github.com/ethz-asl/libpointmatcher).

#### Pre-processing

As a first step, all images were corrected for background fluorescence. A target value for the allowed number of maxima was determined based upon the area of the slice in µm^2^ multiplied by the factor 0.5. This factor was empirically optimized. The brightest maxima per plane were determined, based upon an empirically optimized threshold. The number and location of the respective maxima were stored. This procedure was performed for every image slice independently. Maxima that did not have a neighboring maximum in an adjacent slice (called z-group) were excluded. The resulting maxima list was further filtered in an iterative loop by adjusting the allowed thresholds for Babs-Bback and Bperi-Bback to reach a feature target value (Babs, absolute brightness; Bback, local background; Bperi, background of periphery within one pixel). This feature target values were based upon the volume of the three-dimensional (3D) image. Only maxima still in a group of at least two after filtering passed the filter step. Each z-group was counted as one hit. The members of the z-groups with the highest absolute brightness were used as features and written to a file. They resemble a 3D point cloud. Final signal segmentation and decoding: to align the raw data images from different imaging rounds, images had to be corrected. To do so, the extracted feature point clouds were used to find the transformation matrices. For this purpose, an iterative closest point cloud algorithm was used to minimize the error between two point clouds. The point clouds of each round were aligned to the point cloud of round one (reference point cloud). The corresponding point clouds were stored for downstream processes. Based upon the transformation matrices, the corresponding images were processed by a rigid transformation using tri-linear interpolation. The aligned images were used to create a profile for each pixel consisting of 16 values (16 images from two color channels in eight imaging rounds). The pixel profiles were filtered for variance from zero normalized by total brightness of all pixels in the profile. Matched pixel profiles with the highest score were assigned as an ID to the pixel. Pixels with neighbors having the same ID were grouped. The pixel groups were filtered by group size, number of direct adjacent pixels in group and number of dimensions with size of two pixels. The local 3D maxima of the groups were determined as potential final transcript locations. Maxima were filtered by the number of maxima in the raw data images where a maximum was expected. Remaining maxima were further evaluated by the fit to the corresponding code. The remaining maxima were written to the results file and considered to resemble transcripts of the corresponding gene. The ratio of signals matching to codes used in the experiment and signals matching to codes not used in the experiment was used as estimation for specificity (false positives).

#### Downstream analysis

Initial image analysis was performed in ImageJ using the Polylux tool plugin from Resolve Biosciences to examine specific Molecular Cartography signals.

Because WGA and DAPI staining are difficult to use for segmentation of individual cells within the heart, which span up to 100 µm in size in cardiomyocytes, for downstream analysis, we used a binning approach. *x* and *y* coordinates for each ROI were binned to the nearest 50-pixel (~7 µm) centroid to predict subcellular level of gene co-expression. An AnnData object was generated for each ROI binned matrix and concatenated to generate a merged AnnData object comprising data from all ROIs. Bins were filtered for more than one gene and more than one count per bin (Extended Data Fig. 2c).

For gene correlation analysis, vasculature bins were collectively subset by *VWF*, *MYH11* or *KCNJ8* > 0 counts and TTN = 0 counts. Gene correlation analysis was performed using Pearson correlation and visualized using the seaborn clustermap function^129^. To determine gene expression in vessels of different caliber, we used the Scikit-learn agglomerative hierarchical clustering function using linkage distance to group bins into individual vessels^130^. To determine arterial EC identity, subsetting of binned data was performed using co-expression of pan-EC marker *VWF* and arterial EC marker *GJA5* with ≥1 count per bin. To determine venous identity, subsetting of binned data was performed using pan-EC marker *VWF* and venous EC marker *ACKR1* with ≥1 count per bin. Clusters comprising fewer than five bins were removed. To determine capillary identity, subsetting of binned data was performed using co-expression of pan-EC marker *VWF* and capillary EC marker *RGCC* with ≥1 count per bin.

### Statistics and reproducibility

No statistical method was used to predetermine sample size for scRNA-seq/snRNA-seq analysis. Organs removed from the final scRNA-seq dataset (originating from the Tabula Sapiens dataset^6^) included eye, mammary, prostate, salivary gland, skin and tongue, due to low number of vascular cells and/or skewed donor contribution. The experiments were not randomized. The investigators were not blinded to allocation during experiments and outcome assessment.

Multiplexed smFISH experiments (Figs. 2d,l and 3e and Extended Data Fig. 2a,b) were performed across eight tissue sections from one donor. smFISH experiments for *TINAGL1*, *EGFL7* and *MYLK* (Fig. 1d and Extended Data Fig. 1a) were performed on three donors. smFISH experiments for *ELN*, *SULF1* and *NTS* (Extended Data Figs. 3a,b and 4d) were performed on two donors. Rarecyte experiments for CD8A (Extended Data Fig. 4f) were performed on one donor. Spatial transcriptomics experiments on uterus tissue (Extended Data Fig. 5c) were performed on two tissue sections from two donors.

### Reporting summary

Further information on research design is available in the Nature Portfolio Reporting Summary linked to this article.

## Online content

Any methods, additional references, Nature Portfolio reporting summaries, source data, extended data, supplementary information, acknowledgements, peer review information; details of author contributions and competing interests; and statements of data and code availability are available at 10.1038/s41591-024-03376-x.

## Supplementary information

Supplementary Information Supplementary Figs. 1–8.

Reporting Summary

Supplementary Tables 1–10.

## Data Availability

All unpublished transcriptomic data from this atlas are available through the European Nucleotide Archive (ENA) (https://www.ebi.ac.uk/ena/browser/view/ERP165258). Publicly available datasets used for this study are documented in Supplementary Tables 1 and 8. All relevant processed single-cell and Visium objects, as well as an interactive platform for exploring these and ligand–receptor interaction data, are available through https://www.vascularcellatlas.org/. Publicly available datasets used for this study are documented in Supplementary Tables 1 and 8 and include the following: scRNA-seq: Litviňuková et al.^13^ ((HCA) Data Coordination Platform (DCP) with accession number ERP123138); Madissoon et al.^24^ (ENA under accession number PRJEB52292 and BioStudies accession number S-SUBS17); Kedlian et al. 2024 (ArrayExpress, E-MTAB-13874); Vento-Tormo et al. 2018 (ArrayExpress, E-MTAB-6701); Garcia-Alonso et al.^121^ (ArrayExpress, E-MTAB-10287); Elmentaite et al. 2021 (ArrayExpress, E-MTAB-9543, E-MTAB-9536, E-MTAB-9532, E-MTAB-9533 and E-MTAB-10386); Stewart et al. 2019 (Human Cell Atlas Data Portal, https://data.humancellatlas.org/explore/projects/abe1a013-af7a-45ed-8c26-f3793c24a1f4); Madissoon et al. 2019 (Human Cell Atlas Data Coordination Platform and National Center for Biotechnology Information (NCBI) BioProject accession code PRJEB31843); Brazovskaja et al. 2021 (https://data.mendeley.com/datasets/yp3txzw64c/1); Winkler et al. 2022 (database of Genotypes and Phenotypes (dbGAP), phs002624.v2.p1); Dominguez Conde et al. 2022 (ArrayExpress, E-MTAB-11536); the Tabula Sapiens Consortium, 2022 (Gene Expression Omnibus (GEO), GSE201333). snRNA-seq: Litviňuková et al.^13^ ((HCA) DCP, ERP123138); Madissoon et al.^24^ (ENA, PRJEB52292, and BioStudies accession number S-SUBS17); Perez et al. 2022 (GEO, GSE167186); Lake et al. 2021 (https://portal.hubmapconsortium.org/); Andrews et al. 2022 (GEO, GSE185477); Tosti et al. 2021 (European Genome-Phenome Archive (EGA), EGAS00001004653); Garcia et al.^98^ (GEO, GSE173731); Sun et al. 2023 (https://www.synapse.org/Synapse:syn51015750); and Yang et al. 2022 (GEO, GSE163577).

## Code availability

The ChEMBL database used for drug2cell analysis is available at the following FTP site: https://ftp.ebi.ac.uk/pub/databases/chembl/ChEMBLdb/releases/chembl_30. The drug2cell Python package is available at GitHub (https://github.com/Teichlab/drug2cell). The CellPhoneDB database version 4.1.0 used for interaction analysis is available at https://www.cellphonedb.org/. The custom code for the other analyses performed in this study are available at GitHub (https://github.com/Teichlab/vascular_atlas and https://github.com/NosedaLab/vascular_atlas).
