# Supplementary material for: An organotypic atlas of human vascular cells
Source: Nat Med. 2024 Nov 20;30(12):3468–81. doi: 10.1038/s41591-024-03376-x (PMC11645277; doi:10.1038/s41591-024-03376-x)
Supplement: Supplementary file 1 — Supplementary Figs. 1–8. [file 41591_2024_3376_MOESM1_ESM.pdf]

nature medicine

Supplementary information https://doi.org/10.1038/s41591-024-03376-x

# An organotypic atlas of human vascular cells

In the format provided by the authors and unedited

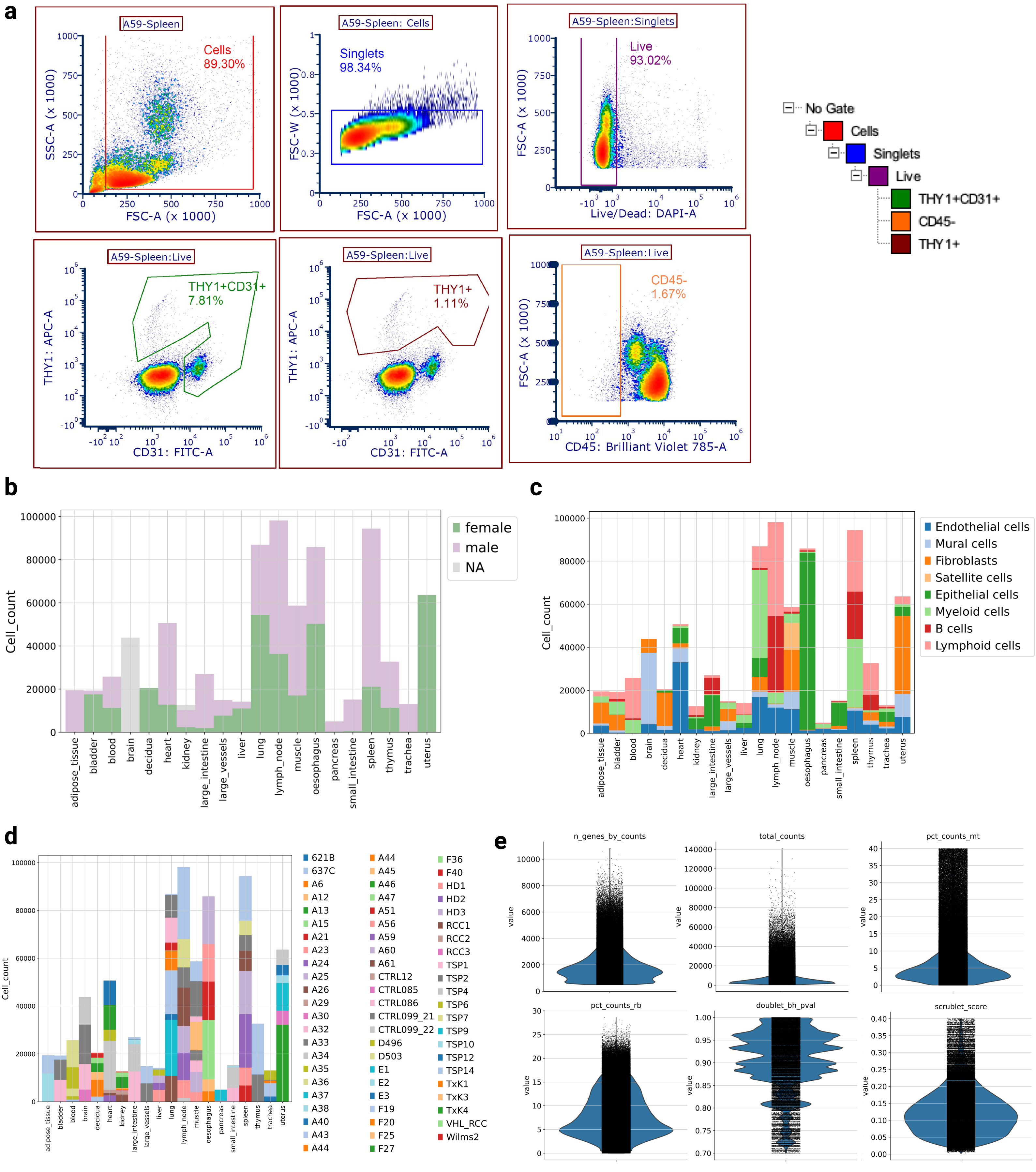


**Supplementary Figure 1. Donor, cell type and sex compositions across datasets. a,** FACS plots of gating strategy for spleen and lymph node cell sorting. **b,** Stacked barplot representing the number of cells from females and males for each organ. **c,** Stacked barplot representing the number of indicated cell types in each organ. **d,** Stacked barplot representing the percentage contribution of cells from indicated donors to each organ. **e,** Violin plots representing QC metrics such as n_genes_by_counts, total_counts, percentage of mitochondrial genes (pct_counts_mt), percentage of ribosomal genes (pct_counts_rb), Benjamini-Hochberg corrected p values for doublet detection (doublet_bh_pval), and scrublet_score for doublet detection.

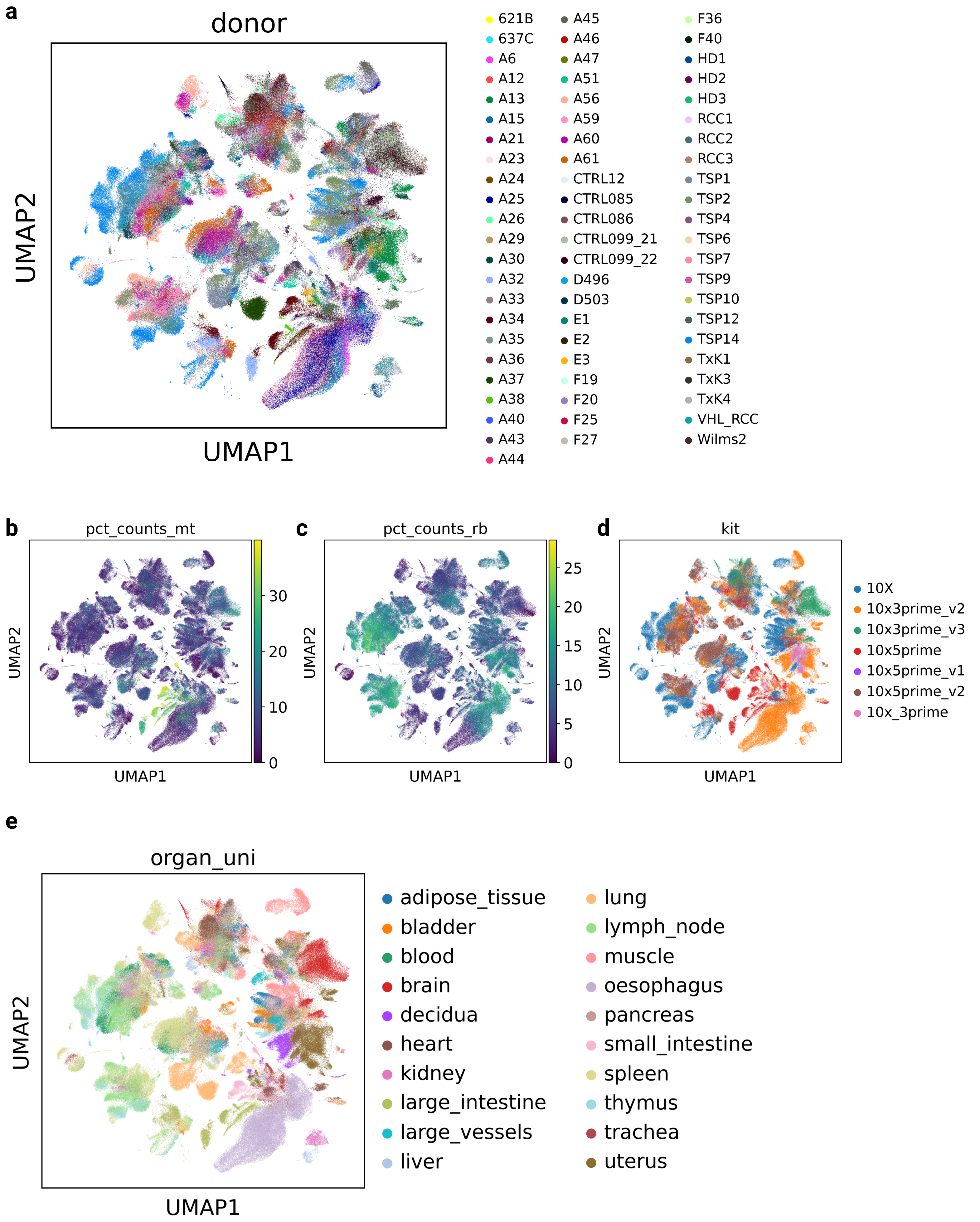


**Supplementary Figure 2. Integration of all cells across all datasets.** UMAP representation of **a,** donors, **b,** percentage mitochondrial content, **c,** percentage ribosomal content, **d,** 10x kit chemistry, and **e,** organs, following integration of all cells in the global object.

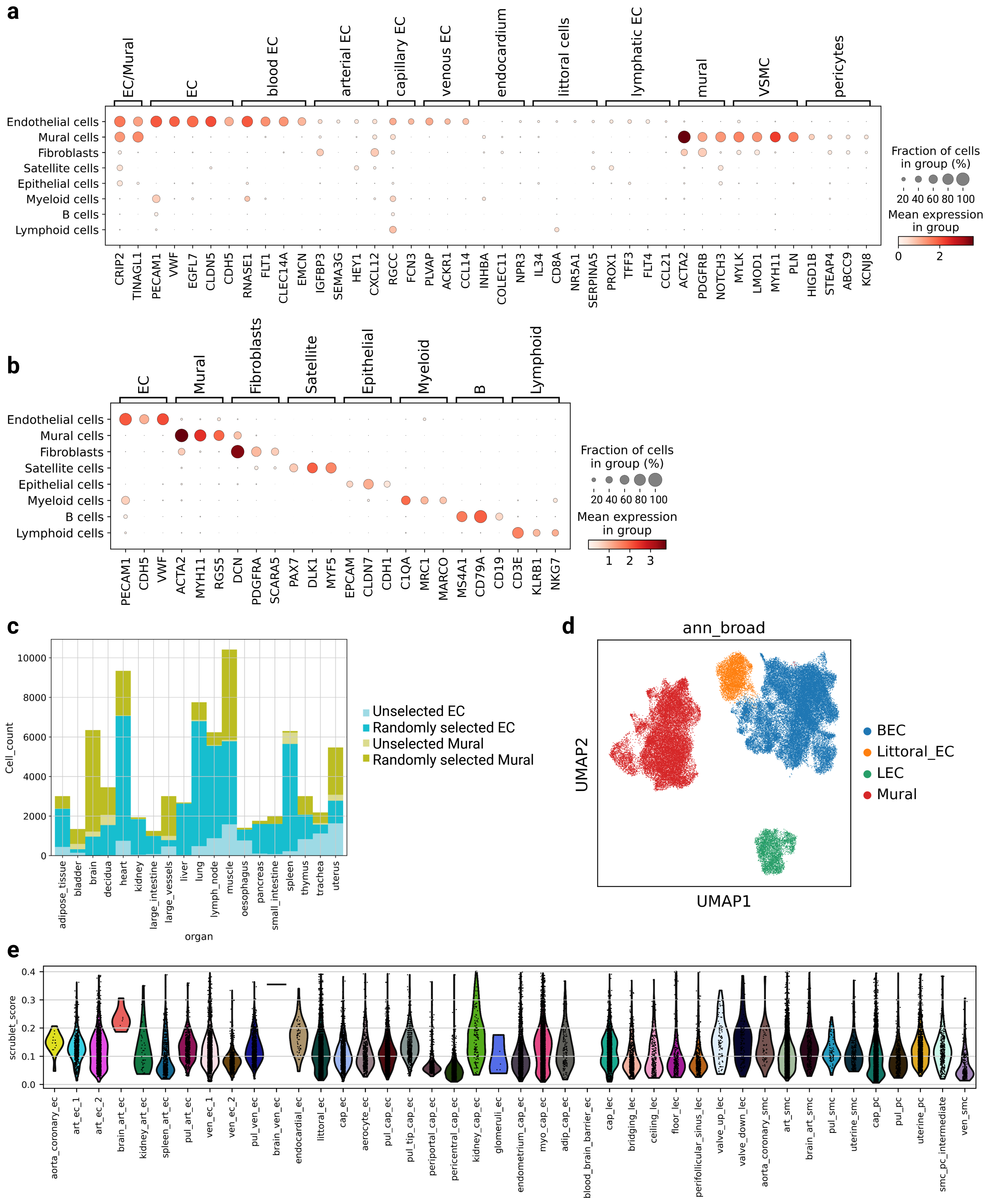


**Supplementary Figure 3. Marker gene expression of endothelial and mural cells. a,** Dotplot representation of endothelial and mural marker genes across global cell states identified through integration of 18 organs, corresponding to Figure 1B. Fraction of expressing cells and average expression within each celltype is indicated by dot size and colour, respectively. **b,** Dotplot representation of cell type markers in global all cell object. Fraction of expressing cells and average expression within each celltype is indicated by dot size and colour, respectively. **c,** Barplot representation of downsampled cells across organs. **d,** UMAP representation of broad cell type level annotation within the vascular compartment relevant for Supplementary Figure 4. **e,** Violin plot representation of scrublet scores for vascular cell states.

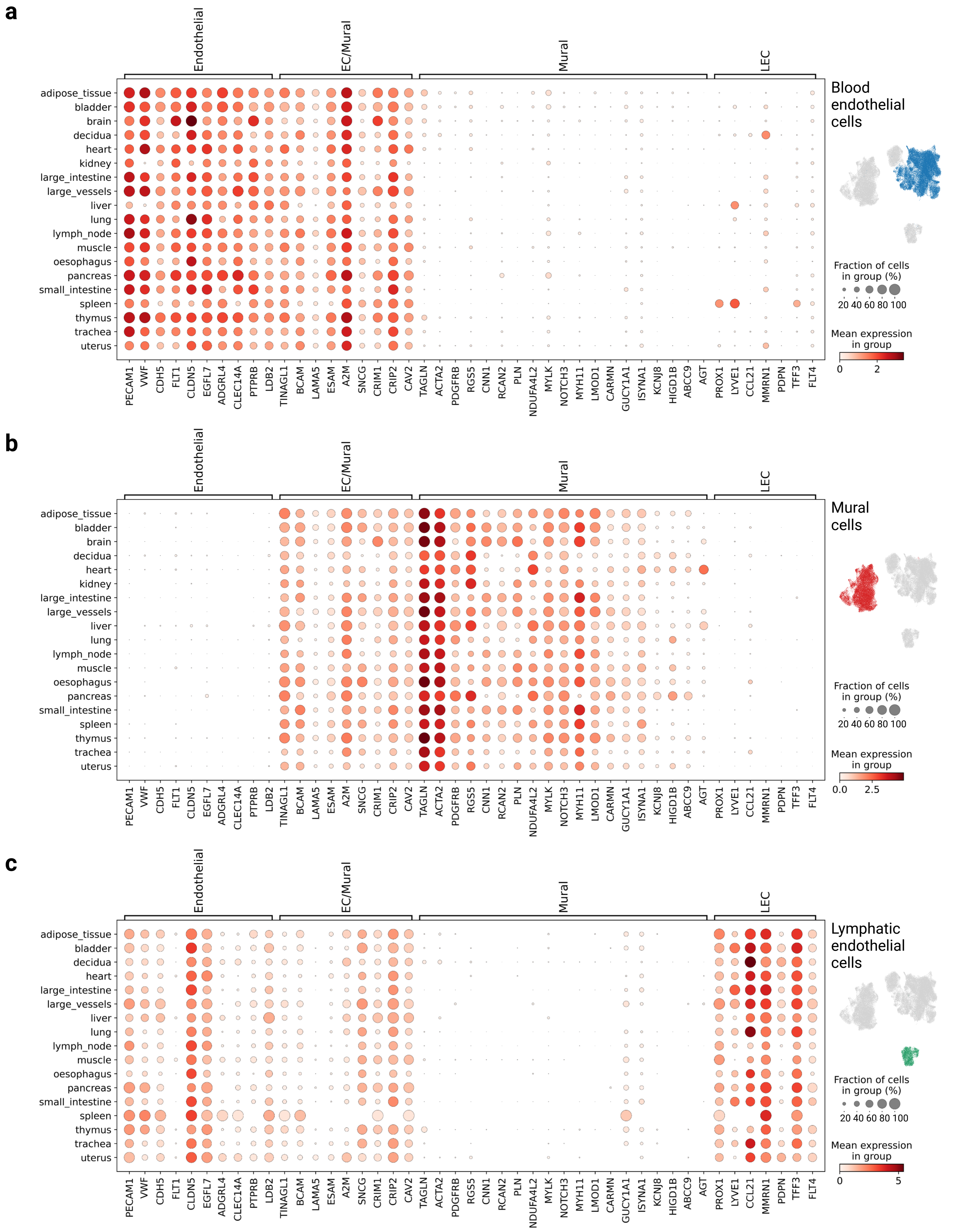


**Supplementary Figure 4. EC, mural, and LEC markers across organs.** Dotplots illustrating the expression of genes for EC, EC/mural, mural and lymphatic ECs across organs in **a,** blood EC, **b,** mural cells, and **c,** lymphatic EC. Fraction of expressing cells and average expression within each organ indicated by dot size and colour, respectively.

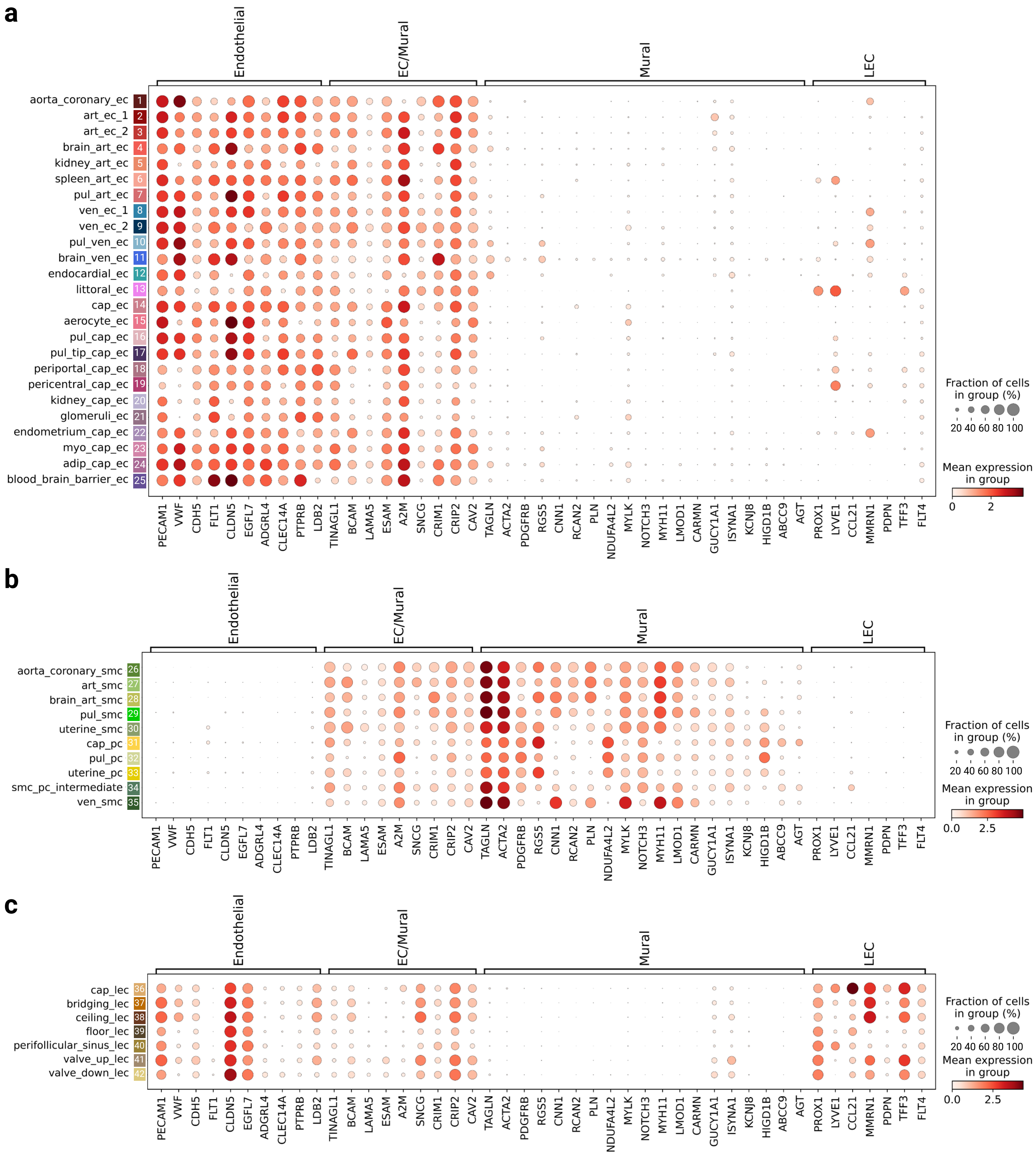


**Supplementary Figure 5. Pan-EC, pan-mural, and pan-LEC markers across cell states. a,** Dotplot representation of pan markers across blood EC cell states. **b,** Dotplot representation of pan markers across mural cell states. **c,** Dotplot representation of pan markers across lymphatic EC cell states. Fraction of expressing cells and average expression within each celltype is indicated by dot size and colour, respectively.

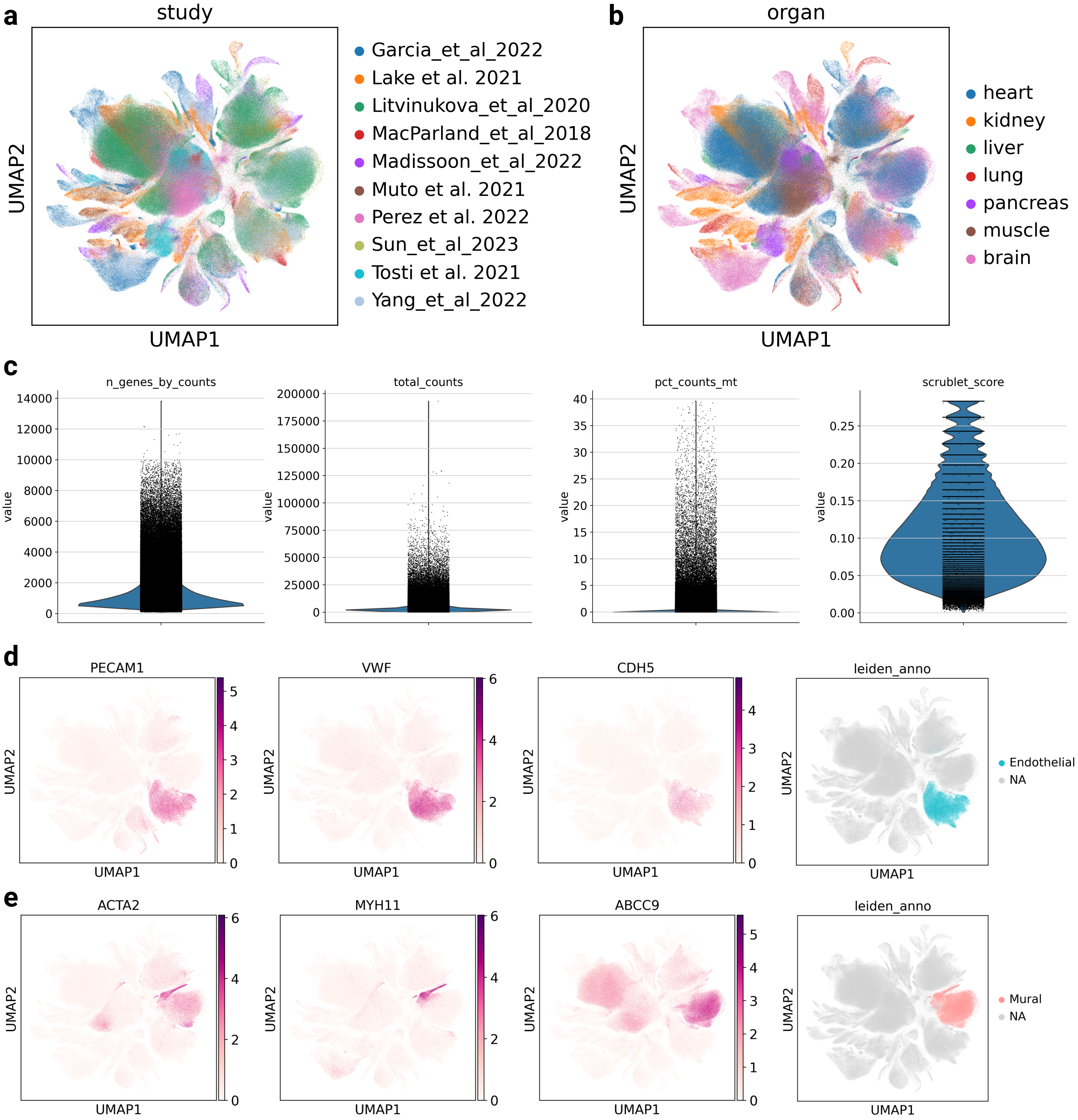


**Supplementary Figure 6. Integration of single-nucleus RNA-seq datasets. a,** UMAP representation of single-nucleus datasets used in the global single-nucleus object. **b,** UMAP representation of organs used in the global single-nucleus object. **c,** Violin plot representation of QC metrics number of genes by counts, total counts, percentage mitochondrial gene counts, and scrublet score. **d,** UMAP representation of EC marker genes *PECAM1*, *VWF*, and *CDH5*, and annotated endothelial cell cluster. **e,** UMAP representation of mural cell markers *ACTA2*, *MYH11* and *ABCC9*, and annotated mural cell cluster.

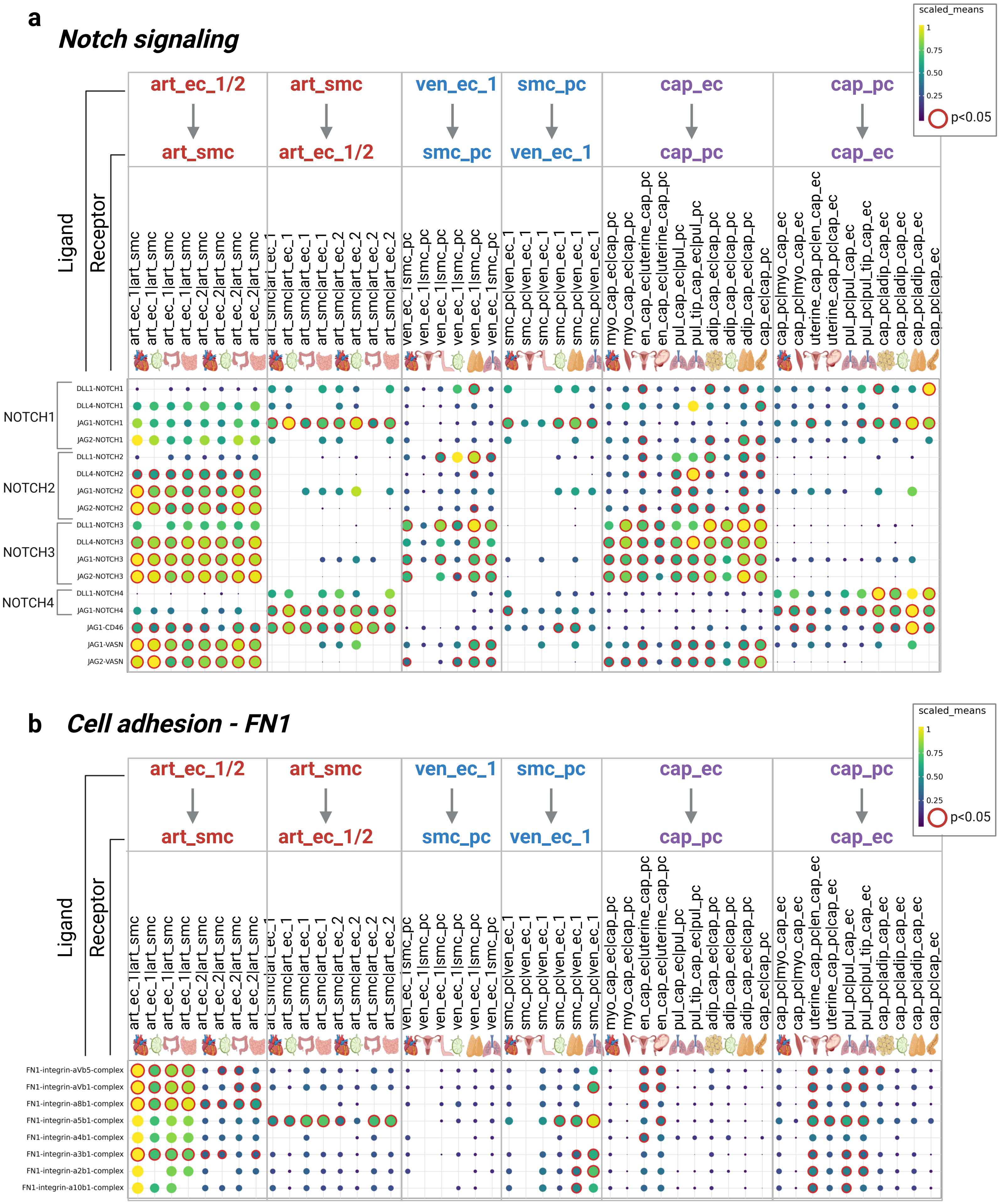


**Supplementary Figure 7. Organotypic intercellular signalling between endothelial and mural cells.** Ligand-receptor (LR) interactions belonging to the NOTCH **(a)**, and the cell adhesion-fibronectin signalling (**b**) between EC and mural cells in pre-defined spatial microenvironments (artery, vein, microvasculature). Specific LR pairs represented on the Y axis. Dot size and colour represent the scaled average gene expression of LR pairs, red highlighting underlines significant interactions with p-values < 0.05. The illustrations in **a** and **b** were created with BioRender.com

# Cell adhesion - collagen

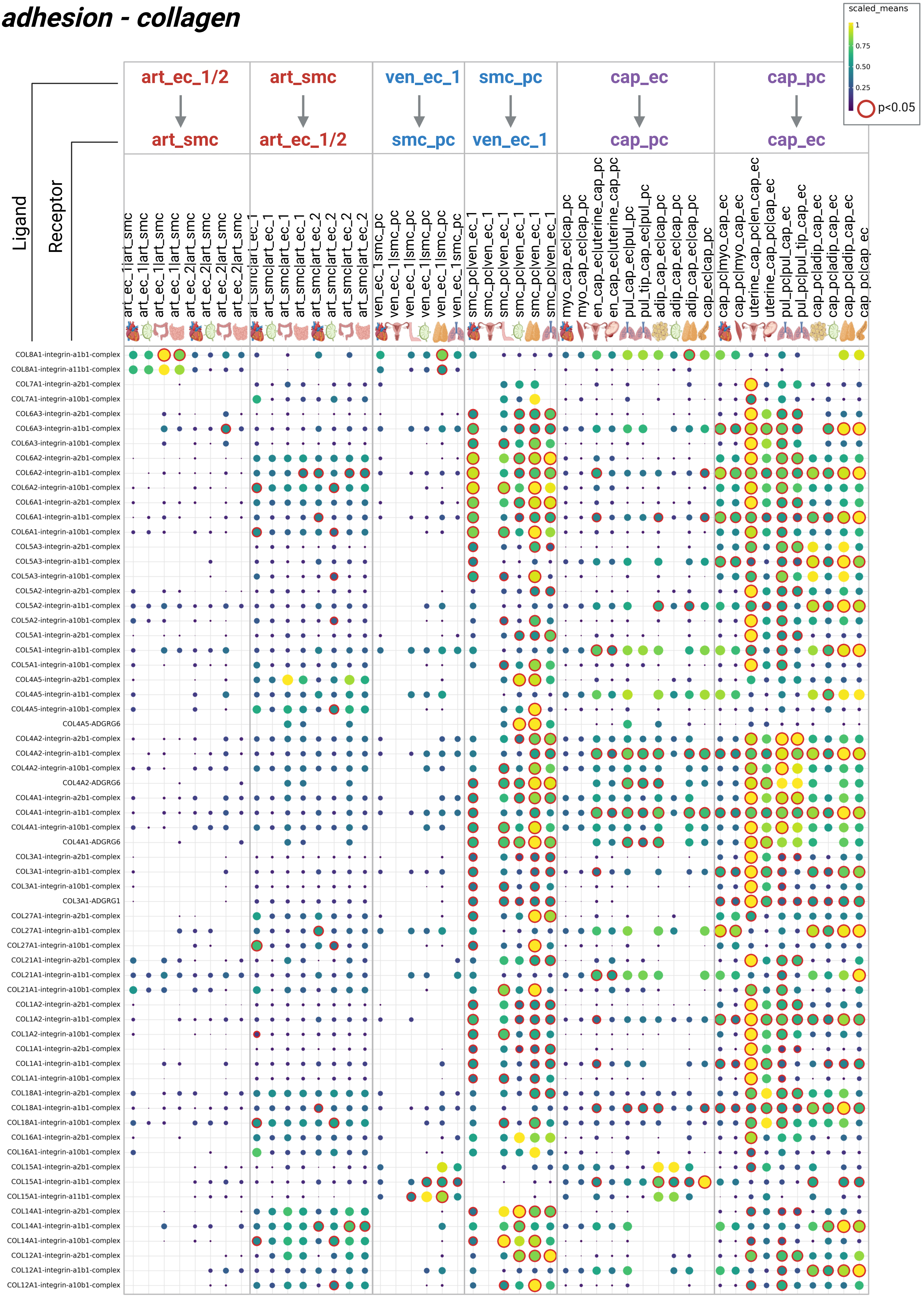


**Supplementary Figure 8. Organotypic intercellular signalling between endothelial and mural cells.** Ligand-receptor (LR) interactions belonging to the cell adhesion-collagen signalling between EC and mural cells in pre-defined spatial microenvironments (artery, vein, microvasculature). Specific LR pairs represented on the Y axis. Dot size and colour represent the scaled average gene expression of LR pairs, red highlighting underlines significant interactions with p-values < 0.05. Illustrations were created with BioRender.com
