# Supplementary material for: An organotypic atlas of human vascular cells
Source: Nat Med. 2024 Nov 20;30(12):3468–81. doi: 10.1038/s41591-024-03376-x (PMC11645277; doi:10.1038/s41591-024-03376-x)
Supplement: Supplementary file 2 — Reporting Summary [file 41591_2024_3376_MOESM2_ESM.pdf]

nature portfolio

Corresponding author(s): Michela Noseda
Sarah Teichmann

Last updated by author(s): Oct 21, 2024

Reporting Summary

Nature Portfolio wishes to improve the reproducibility of the work that we publish. This form provides structure for consistency and transparency in reporting. For further information on Nature Portfolio policies, see our Editorial Policies and the Editorial Policy Checklist.

Statistics

For all statistical analyses, confirm that the following items are present in the figure legend, table legend, main text, or Methods section.

n/a	Confirmed	
☐	☒	The exact sample size (*n*) for each experimental group/condition, given as a discrete number and unit of measurement
☐	☒	A statement on whether measurements were taken from distinct samples or whether the same sample was measured repeatedly
☐	☒	The statistical test(s) used AND whether they are one- or two-sided<br>*Only common tests should be described solely by name; describe more complex techniques in the Methods section.*
☐	☒	A description of all covariates tested
☐	☒	A description of any assumptions or corrections, such as tests of normality and adjustment for multiple comparisons
☐	☒	A full description of the statistical parameters including central tendency (e.g. means) or other basic estimates (e.g. regression coefficient) AND variation (e.g. standard deviation) or associated estimates of uncertainty (e.g. confidence intervals)
☐	☒	For null hypothesis testing, the test statistic (e.g. *F*, *t*, *r*) with confidence intervals, effect sizes, degrees of freedom and *P* value noted<br>*Give P values as exact values whenever suitable.*
☒	☐	For Bayesian analysis, information on the choice of priors and Markov chain Monte Carlo settings
☒	☐	For hierarchical and complex designs, identification of the appropriate level for tests and full reporting of outcomes
☐	☒	Estimates of effect sizes (e.g. Cohen's *d*, Pearson's *r*), indicating how they were calculated

Our web collection on statistics for biologists contains articles on many of the points above.

Software and code

Policy information about availability of computer code

Data collection	Software used include: Single cells were sorted using the Sony MA900 and SH800 sorters and their proprietary software (Cell Sorter Software v3.1.1). Single cell data were aligned using 10x Genomics CellRanger (v3.0) and STARsolo (v2.7.3a). Visium spatial transcriptomics samples were aligned using 10x Genomics SpaceRanger (v1.1.0).
Data analysis	Single cell data analysis and plotting was performed using Python (v3), scanpy (v1.8.2 and v1.9.1), anndata (v0.8.0 and 0.9.1), numpy (v1.21.6 and v1.23.5), scipy (v1.8.0 and 1.9.3), pandas (v1.5.2 and v1.5.3), seaborn (v0.12.2), matplotlib (v3.5.2), cellphonedb (v4.0.0) with database v4.1.0, drug2cell (v0.1.0) with ChEMBL v30, pySCENIC (0.11.2). Ambient RNA removal was performed using cellbender (v0.2.0). Doublet removal was performed using scrublet (v0.2.3). Batch correction using scVI (v0.9.1). Spatial mapping was performed using cell2location (v0.1.3). RNAscope image quantification was performed in Fiji (v2.14.0). Additional custom codes used in this manuscript is available at https://github.com/Teichlab/vascular_atlas. BioRender was used to generate schematics (as described in manuscript figure legends).

For manuscripts utilizing custom algorithms or software that are central to the research but not yet described in published literature, software must be made available to editors and reviewers. We strongly encourage code deposition in a community repository (e.g. GitHub). See the Nature Portfolio guidelines for submitting code & software for further information.

Data

Policy information about availability of data

All manuscripts must include a data availability statement. This statement should provide the following information, where applicable:

- Accession codes, unique identifiers, or web links for publicly available datasets
- A description of any restrictions on data availability
- For clinical datasets or third party data, please ensure that the statement adheres to our policy

All unpublished single cell transcriptomic data from this atlas are available through ENA European Nucleotide Archive (https://www.ebi.ac.uk/ena/browser/view/ERP165258).

The human reference genome (GRCh38) used for read mapping is available from the 10x Genomics website (https://support.10xgenomics.com/single-cell-gene-expression/software/release-notes/build).

All relevant processed single cell and Visium objects, as well as an interactive platform for exploring these and ligand-receptor interactions data, are available through https://www.vascularcellatlas.org/ (Username: vascular; Password: vasc1atlas).
The ChEMBL database used for drug2cell analysis is available from https://ftp.ebi.ac.uk/pub/databases/chembl/ChEMBLdb/releases/chembl_30.
The drug2cell Python package is available at GitHub (https://github.com/Teichlab/drug2cell).
The CellPhoneDB database version 4.1.0 used for interaction analysis is available at https://www.cellphonedb.org/.

Publicly available datasets used for this study are documented in Table S1 and S8, and include:
scRNA-seq - Litviňuková et al. 2020 (Human Cell Atlas Data Coordination Platform (DCP) with accession number: ERP123138); Madissoon et al. 2022 (European Nucleotide Archive (ENA) under accession number PRJEB52292 and BioStudies accession S-SUBS17); Kedlian et al. 2024 (ArrayExpress (E-MTAB-13874)); Vento-Tormo et al. 2018 (ArrayExpress, E-MTAB-6701); Garcia-Alonso et al. 2021 (ArrayExpress under accession numbers E-MTAB-10287); Elmentaite et al. 2021 (ArrayExpress with accession numbers E-MTAB-9543, E-MTAB-9536, E-MTAB-9532, E-MTAB-9533 and E-MTAB-10386); Stewart et al. 2019 (Human Cell Atlas Data Portal (https://data.humancellatlas.org/explore/projects/abe1a013-af7a-45ed-8c26-f3793c24a1f4); Madissoon et al. 2019 (Human Cell Atlas Data Coordination Platform and NCBI BIOPROJECT accession code PRJEB31843); Brazovskaja et al. 2021 (https://data.mendeley.com/datasets/yp3txzw64c/1); Winkler et al. 2022 (dbGAP phs002624.v2.p1); Dominguez Conde et al. 2022 (www.ebi.ac.uk/arrayexpress under accession number E-MTAB-11536); The Tabula Sapiens Consortium, 2022 (Gene Expression Omnibus with accession number GSE201333).
snRNA-seq - Litviňuková et al. 2020 (Human Cell Atlas Data Coordination Platform (DCP) with accession number: ERP123138); Madissoon et al. 2022 (European Nucleotide Archive (ENA) under accession number PRJEB52292 and BioStudies accession S-SUBS17); Perez et al. 2022 (Gene Expression Omnibus (GEO) GSE167186); Lake et al. 2021 (https://portal.hubmapconsortium.org/); Andrews et al. 2022 (GEO under accession: GSE185477); Tosti et al. 2021 (https://www.ebi.ac.uk/ega/home under EGAS00001004653); Garcia et al. 2022 (NCBI GEO under accession GSE173731.); Sun et al. 2023 (https://www.synapse.org/Synapse:syn51015750); Yang et al. 2022 (NCBI Gene Expression Omnibus (GEO) under accession code GSE163577).

Research involving human participants, their data, or biological material

Policy information about studies with human participants or human data. See also policy information about sex, gender (identity/presentation), and sexual orientation and race, ethnicity and racism.

Reporting on sex and gender	Spleen tissue used for scRNA-seq was obtained from 3 male donor (A51, A59, A60), and 1 female donor (A61).<br>Lymph node tissue used for scRNA-seq was obtained from 1 female donor (A16).<br>Skeletal muscle used for RNAscope was obtained from 1 female donor (A66), and 1 male donor (A60).<br>Ileum tissue used for RNAscope was obtained from 1 male donor (A64) and one female donor (A74).<br>Cardiac tissue used for multiplex-smFISH (Molecular Cartography) was obtained from 1 female donor (AH6).<br><br>Sex-related analysis was not performed due to a lack of sufficient sample numbers across donors.
Reporting on race, ethnicity, or other socially relevant groupings	Race, ethnicity, or other socially relevant groupings are not reported or analysed in this study.
Population characteristics	Heart, spleen, lymph node, skeletal muscle, and ileum tissues were collected in the United Kingdom. All samples are from adult individuals. A16: female, 20-25. A51: male, age 35-40. A59: male, age 45-50. A60: male, age 50-55. A61: female, age 70-75. A64: male, adult. A66: female, age 20-25. A74: female, adult. AH6: female, age 50-55
Recruitment	Tissues were obtained from healthy transplant organ donors. Heart, spleen, lymph node, skeletal muscle and ileum tissue were retrieved from donors by trained surgeons using clinical transplant retrieval protocols. We believe this method of recruitment does not introduce any bias that can impact our results.
Ethics oversight	Human ileum, lymph node, spleen and skeletal muscle tissues were obtained from Cambridge Biorepository for Translational Medicine (CBTM) under the agreement: REC reference 15/EE/0152, East of England Cambridge South Research Ethics Committee and approved by National Institute for Health and Care Research (NIHR203312).<br><br>Human heart samples (AH6) were obtained from deceased tranplant organ donors after Research Ethics Committee approval and informed consent from the donor families. Human heart samples used for multiplex-smFISH and Visium experiments were acquired by Imperial College London under the agreement: REC reference 16/LO/1568, London, London Bridge Research Ethics Committee.

Note that full information on the approval of the study protocol must also be provided in the manuscript.

Field-specific reporting

Please select the one below that is the best fit for your research. If you are not sure, read the appropriate sections before making your selection.

☒ Life sciences ☐ Behavioural & social sciences ☐ Ecological, evolutionary & environmental sciences

For a reference copy of the document with all sections, see nature.com/documents/nr-reporting-summary-flat.pdf

Life sciences study design

All studies must disclose on these points even when the disclosure is negative.

Sample size	Sample size was determined based on the availability of donors, and availability of publicly available scRNA-seq data. No statistical methods were used to calculate appropriate sample size. We follow standards in the field and Human Cell Atlas criteria.
Data exclusions	Low-quality cells were filtered out by the following criteria: minimum number of reads = 500, minimum number of genes = 300, percentage of mitochondrial genes <= 0.4, percentage of ribosomal genes <= 0.3, scrublet score <= 0.4, p value of gb corrected scrublet score >= 0.7. After vascular cell subsetting, samples were downsampled to a maximum of 1,500 cells per donor per organ.<br><br>Organs removed from the final dataset (originating from Tabula sapiens data) included eye, mammary, prostate, salivary gland, skin and tongue, due to low number of vascular cells and / or skewed donor contribution.
Replication	Single cell RNA-seq data was obtained from 4 spleen donors and 4 lymph node donors. Visium spatial transcriptomics was performed across 2 regions of 2 donor hearts, with all attempts successful.<br>SULF1 and ELN RNAscope experiments were performed on 2 donor samples, with both attempts successful.<br>TINAGL1, EGFL7 and MYLK RNAscope experiments were performed on 3 donor samples, with all attempts successful.<br>NTS RNAscope experiments were performed on 2 donor samples, with both attempts successful.<br>CD8A Rarecyte experiments were performed on 1 donor and not replicated.<br>Multiplexed smFISH (Molecular CartographyTM) experiments were performed on 8 sections from 1 donor and not replicated.
Randomization	Randomization was not relevant due to the study design where sample collection was based on availability of transplant donors, and there were no experimental groups to be compared, i.e. healthy vs. disease cohorts, in this dataset.
Blinding	For the sequencing samples, we made no comparison between discrete groups for human participants. There were also no experimental groups to be compared, i.e. healthy vs. disease cohorts, in this dataset. Thus, blinding was not necessary

Reporting for specific materials, systems and methods

We require information from authors about some types of materials, experimental systems and methods used in many studies. Here, indicate whether each material, system or method listed is relevant to your study. If you are not sure if a list item applies to your research, read the appropriate section before selecting a response.

Materials & experimental systems

n/a	Involved in the study	
☐	☒	Antibodies
☒	☐	Eukaryotic cell lines
☒	☐	Palaeontology and archaeology
☒	☐	Animals and other organisms
☒	☐	Clinical data
☒	☐	Dual use research of concern
☒	☐	Plants

Methods

n/a	Involved in the study	
☒	☐	ChIP-seq
☐	☒	Flow cytometry
☒	☐	MRI-based neuroimaging

Antibodies

Antibodies used	Primary antibodies:<br>Mouse Anti-CD144 (VE-Cadherin), IgG1 (ThermoFisher Scientific - Cat#14-1449-82, clone#16B1, monoclonal, dilution 1:3000)<br>Mouse Anti-alpha smooth muscle actin (ACTA2), IgG2a (Abcam - Cat# ab7817, clone#1A4, monoclonal, dilution 1:3000)<br><br>Secondary antibodies:<br>Goat anti-Mouse IgG1 (ThermoFisher Scientific - Cat#A10551, polyclonal, dilution 1:1000)<br>Goat anti-Mouse IgG2a (ThermoFisher Scientific - Cat#A10685, polyclonal, dilution 1:1000).<br><br>Conjugated antibodies:<br>CD31 (FITC anti-human CD31, WM59 clone, Bd Biosciences #555445, dilution 1:50)<br>CD45 (BV785 anti-human CD45, HI30 Clone (mouse), Biolegend #304048, dilution 1:50)<br>PDPN (PE anti-human PDPN, NC-08 clone, Biolegend #337003, dilution 1:50)

THY1 (APC anti-human CD90(THY1), 5E10 clone (mouse), Bd Biosciences #561971, dilution 1:50)

Nuclei were stained with DAPI (ThermoFisher Scientific - Cat#D1306).

Validation	Mouse Anti-CD144 (VE-Cadherin) validated by manufacturer for Western blot, immunohistochemistry, immunocytochemistry, flow cytometry.<br>Mouse Anti-alpha smooth muscle actin validated by manufacturer for Western blot, immunohistochemistry, immunocytochemistry, flow cytometry<br>FITC anti-human CD31 validated by manufacturer for flow cytometry<br>BV785 anti-human CD45 validated by manufacturer for flow cytometry<br>PE anti-human PDPN validated by manufacturer for flow cytometry<br>APC anti-human CD90 (THY1) validated by manufacturer for flow cytometry

Plants

Seed stocks	n/a
Novel plant genotypes	n/a
Authentication	n/a

Flow Cytometry

Plots

Confirm that:

☒ The axis labels state the marker and fluorochrome used (e.g. CD4-FITC).

☒ The axis scales are clearly visible. Include numbers along axes only for bottom left plot of group (a 'group' is an analysis of identical markers).

☒ All plots are contour plots with outliers or pseudocolor plots.

☒ A numerical value for number of cells or percentage (with statistics) is provided.

Methodology

Sample preparation	Relevant to unpublished spleen and lymph node data only. Upon removal, spleen / lymph node samples were transferred into Hypothermosol (Sigma-adrich H4416-100ML) and shipped to Wellcome Sanger Institute on ice within 24 hrs. Samples were washed in DBPS, cleaned from fat and connective tissue, minced and digested in the mixture of liberaseTH (Roche, 05401135001) and DNAse-I (Roche, 4716728001) in RPMI media for 30 min. After 30 min, digestion was stopped with 10% of FBS in RPMI, digested cells were strained through a 70 µm strainer, pelleted at 500g and underwent red blood cell lysis using a buffer from eBioscience™ (00-4333-57). If there was remaining tissue left after 30 min, it was digested with a fresh mixture of liberaseTH and DNAse-I in RPMI for an additional 15-30 mins and underwent RBC. To isolate stromal cells, either magnetic (donor A51) or FACS sorting (donors A59, A60 and A61) was employed. Magnetic sorting to deplete CD45+ cells was performed using a magnetic sorting kit from Miltenyi Biotec including LS Columns (130-042-401) and CD45 MicroBeads, human (130-045-801). For FACS sorting, cells were resuspended in the FACS buffer (0.5% FBS and 2mM EDTA in PBS), underwent blocking in TruStainFcX (422302, Biolegend) for 10 min and were stained with a mixture of CD31 (anti-CD31 FITC, WM59 clone, Bd Biosciences #555445), CD45 (CD45 BV785, HI30 Clone (mouse), Biolegend #304048), PDPN (PE anti-human PDPN, Biolegend #337003) and THY1 (APC anti-human CD90(THY1), 5E10 clone (mouse), Bd Biosciences #561971) antibodies and DAPI for 30 min. After staining, cells were washed and analysed using the Sony SH800 or Sony MA900 sorters with 130 µm nozzle.
Instrument	Sony SH800 or Sony MA900 sorters were used for sorting single cells.
Software	Proprietary software (Cell Sorter Software v3.1.1) of the MA900 sorter and SH800 sorter.
Cell population abundance	As many cells as possible were obtained under the gating strategy outlined below.
Gating strategy	Cells were first gated to remove doublets and dead cells. Cell fractions from spleen and lymph node were then sorted using a combination of THY1+, THY+/CD31+, CD45-, or total cell live fraction (Supplementary Data Figure 1).

☒ Tick this box to confirm that a figure exemplifying the gating strategy is provided in the Supplementary Information.
